# Supplementary material for: The crystal structure of bacteriophage λ RexA provides novel insights into the DNA binding properties of Rex-like phage exclusion proteins
Source: Nucleic Acids Res. 2024 Mar 30;52(8):4659–75. doi: 10.1093/nar/gkae212 (PMC11077077; doi:10.1093/nar/gkae212)
Supplement: gkae212_Supplemental_Files [file gkae212_supplemental_files.zip › Rexa_supplemental_NAR_rev1_final.pdf]

Supplementary information for:

THE CRYSTAL STRUCTURE OF BACTERIOPHAGE λ REXA PROVIDES NOVEL INSIGHTS INTO THE DNA BINDING PROPERTIES OF REX-LIKE PHAGE EXCLUSION PROTEINS

AUTHORS

Myfanwy C. Adams[1‡], Carl J. Schiltz[1‡], Jing Sun[1], Christopher J. Hosford[1], Virginia M. Johnson[1], Hao Pan[1], Peter P. Borbat[2,3], Jack H. Freed[2,3], Lynn C. Thomason[4], Carolyn Court[4], Donald L. Court[4], and Joshua S. Chappie[1,*]

[1] Department of Molecular Medicine, Cornell University, Ithaca, NY, 14853, USA
[2] Department of Chemistry and Chemical Biology, Cornell University, Ithaca, 14853, USA
[3] National Biomedical Resource for Advanced Electron Spin Resonance Spectroscopy, Cornell University, Ithaca, 14853, USA
[4] Center for Cancer Research, National Cancer Institute, Frederick, MD, 21702, USA

* To whom correspondence should be addressed. Tel: Tel: +1 (607) 253-3654; Fax: +1 (607) 253-3659; Email: chappie@cornell.edu
† These authors contributed equally

Present Address: Myfanwy C. Adams, John Innes Centre, Norwich Research Park, Norwich, UK
Present Address: Carl J. Schilz, IDEXX Laboratories, Westbrook, ME, 04092, USA
Present Address: Jing Sun, Harvard University, Cambridge, MA 02140, USA
Present Address: Christopher J. Hosford, LifeMine Therapeutics, Cambridge, MA 02140, USA
Present Address: Virginia M. Johnson, Crop Science Division, Bayer, St. Louis, MO, 63141 USA
Present Address: Hao Pan, Sanofi, Waltham, MA 02451, USA

Supplementary Tables

Supplementary Table S1. X-ray data collection and refinement statistics.

Supplementary Table S2. Sequences of oligonucleotide substrates.

Supplementary Table S3. Dissociation constants from EMSA experiments.

Supplementary Table S4. *E. coli* K12 strains used for exclusion and papillation assays.

Supplementary statistical analysis of papillation data

Supplementary Figures

Supplementary Figure S1. Organization and regulation of the λ immunity region. Related to Figures 1 and 6.

Supplementary Figure S2. Location of RexA mutants. Related to Figures 1 and 4.

Supplementary Figure S3. Structural coordination of bound ions and sulfates. Related to Figure 1.

Supplementary Figure S4. Structure and topology of E. coli RdgC. Related to Figure 2.

Supplementary Figure S5. Additional structural homologies identified by Dali. Related to Figures 1, 2, and 3.

Supplementary Figure S6. Sequence alignment of putative RexA homologs. Related to Figures 3, 4, and 5.

Supplementary Figure S7. SEC analysis of RexA mutants. Related to Figures 4, 5, and 6.

Supplementary Figure S8. ESR spectroscopy measurements for D168C. Related to Figure 5.

Supplementary Figure S9. Gene neighborhoods surrounding RexA-like genes in Actinobacteriophages. Related to Figure 7.

Supplementary Figure S10. Sequence alignment of unique RexA-like proteins present in Actinobacteriophage viruses**.** Related to Figure 7.

Supplementary Figure S11. SEC analysis of purified RexA homologs. Related to Figure 7.

Supplementary Figure S12. Comparison of operator sequences used in binding experiments. Related to Figures 4, 5, and 7.

Supplementary Figure S13. AlphaFold model of Toast gp42 and comparison to RexA. Related to Figures 3 and 7.

Supplementary Table S1. X-ray data collection and refinement statistics.

	RexA SeMet<br>Crystal form 1	RexA SeMet<br>Crystal form 2<br>PDB: 8TWQ
**Data collection**		
Space group	P32 2 1	P32 2 1
Cell dimensions		
a, *b*, *c* (Å)	56.4, 56.4, 326.22	55.65, 55.65, 322.12
α, β, γ (°)	90, 90, 120	90, 90, 120
Resolution (Å)	108.74 – 2.68 (2.81 – 2.68)	161.06 – 2.05 (2.11 – 2.05)
R_{sym} or R_{merge}	0.127 (0.913)	0.118 (0.931)
R_{meas}	0.132 (0.997)	0.128 (1.010)
$CC_{1/2}$ (%)	0.997 (0.634)	0.998 (0.595)
$I / \sigma I$	14.2 (1.7)	13.4 (1.9)
Completeness (%)	100.0 (100.0)	99.9 (100.0)
Redundancy	8.9 (8.0)	6.8 (6.6)
**Phasing**		
Initial F.O.M.	0.3	
Number of sites	14	
**Refinement**		
Resolution (Å)		53.69 – 2.05
No. reflections		37768 (3651)
R_{work} / R_{free} (%)		0.2063 / 0.2512
No. atoms		4637
Protein		4367
Ligand/ion		49
Water		221
B-factors		37.46
Protein		37.24
Ligand/ion		58.93
Water		37.18
R.m.s deviations		
Bond lengths (Å)		0.0009
Bond angles (°)		1.04
**Ramachandran statistics**		
Favored (%)		92.86
Allowed (%)		6.04
Outliers (%)		1.10

*Values in parentheses are for highest-resolution shell. Each dataset was derived from a single crystal.

Supplementary Table S2. Sequences of oligonucleotide substrates.

Oligonucleotide	Sequence
EMSA_02_US	5’- CCACTGGCGGTGAT -3’
EMSA_02*_US	5’- (6-FAM) – CCACTGGCGGTGAT -3’
EMSA_02_LS	5’- ATCACCGCCAGTGG -3’
Rex_O_R1-O_R2_US	5’- CATTATCACCGCCAGAGGTAAAATAGTCAACACGCACGGTGTTAGAT -3’
Rex_O_R1-O_R2_LS	5’- ATCTAACACCGTGCGTGTTGACTATTTTACCTCTGGCGGTGATAATG -3’
Rex_O_R1-O_R2_Scram_US	5’- CATGCCAGGATATTCGCTACAAATAGTACGTGATACGACATCGCGAT -3’
Rex_O_R1-O_R2_Scram_LS	5’- ATCGCGATGTCGTATCACGTACTATTTGTAGCGAATATCCTGGCATG -3’
Toast_O_R1-O_R2-O_R3_US	5’-<br>CACCGACCCGGCTACCGCTTAACGCGTGTCGTGTCGCACCTGCCGTCTAC<br>CAATGACTACCGGGGATTGTGCCTCATATCCGCAGGCTGCGGAACTACAG<br>GGCTGTAATTTCTTGACGAGCGTCTACCTGCTGCGGAAGAATCATCCGCA<br>GCCTATTGACACCACCCCGTCTACCAGGAGAGACTCATG -3’
Toast_O_R1-O_R2-O_R3_LS	5 -<br>CATGAGTCTCTCCTGGTAGACGGGGTGGTGTCAATAGGCTGCGGATGATT<br>CTTCCGCAGCAGGTAGACGCTCGTCAAGAAATTACAGCCCTGTAGTTCCG<br>CAGCCTGCGGATATGAGGCACAATCCCCGGTAGTCATTGGTAGACGGCA<br>GGTGCGACACGACACGCGTTAAGCGGTAGCCGGGTCGGTG -3’
Sbash_O_R1-O_R2-O_R3_US	5’-<br>CATACGTCGAGTCTGACAGAAACATTCGGCAAACTTTCGAACTGGGTGGC<br>AAACATCATCAGTGTAATTTCCTCCGACTATTGGGTCTTAGGTCAAGAAAC<br>CCCTGGTAGTCCAGGTTTGCCGTTTTAGGAAAAGTTTCGGCATGTCGGTTT<br>GCCGACCCGAATCTACGGTGTATCTTCGGGGATATG -3’
Sbash_O_R1-O_R2-O_R3_LS	5’-<br>CATATCCCCGAAGATACACCGTAGATTCGGGTCGGCAAACCGACATGCCG<br>AAACTTTTCCTAAAACGGCAAACCTGGACTACCAGGGGTTTCTTGACCTAA<br>GACCCAATAGTCGGAGGAAATTACACTGATGATGTTTGCCACCCAGTTCG<br>AAAGTTTGCCGAATGTTTCTGTCAGACTCGACGTATG -3’
CarolAnn_O_R1-O_R2-<br>O_R3_US	5’-<br>CATGGATTGAGGTTCAACCGCCCGTCCTTCGGCACGCAACGCGCAACGG<br>AACCTATGCGGTTACCGCGTTTGCTCGCTCCGGCTTCCTTCTCCGTGTAC<br>CAACTTGTCCCCCGGATTGTGCCGCATACACGCAGGCTGCGGAACTACCG<br>CCGTGTAATTCATTGCCATGCGTGTACCCACCATGCAAGAATCATACGCAG<br>TTGCTTGACACCCAGCCGGTTACCGAGGAGGATCAATGGCGAGCTACACA<br>CGCTGGCCGAAGGGAAGTTGGATG -3’
CarolAnn_O_R1-O_R2-O_R3_LS	5’-<br>CATCCAACTTCCCTTCGGCCAGCGTGTGTAGCTCGCCATTGATCCTCCTC<br>GGTAACCGGCTGGGTGTCAAGCAACTGCGTATGATTCTTGCATGGTGGGT<br>ACACGCATGGCAATGAATTACACGGCGGTAGTTCCGCAGCCTGCGTGTAT<br>GCGGCACAATCCGGGGGACAAGTTGGTACACGGAGAAGGAAGCCGGAGC<br>GAGCAAACGCGGTAACCGCATAGGTTCCGTTGCGCGTTGCGTGCCGAAG<br>GACGGGCGGTTGAACCTCAATCCATG -3’

Supplementary Table S3. Dissociation constants from EMSA experiments

	K_d (app) μM	+/- Std. Dev.
**Figure 4**		
WT	1.25	0.068
G140A	0.93	0.10
G140P	0.86	0.11
G140-K143>AAAA	ND	ND
Δ239-244	7.94	0.48
D215W	0.14	0.0028
R219A/K221A	ND	ND
C258S/C269S/K2C	1.80	0.0095
**Figure 7A – Lambda Operator (top row)**		
Sbash30	3.50	1.80
CarolAnn44	7.02	1.00
Toast42	9.25	2.88
**Figure 7B – Scrambled Lambda Operator (middle row)**		
Sbash30	5.89	1.28
CarolAnn44	8.84	0.88
Toast42	10.10	2.80
**Figure 7C – Cognate Operator (bottom row)**		
Sbash30	0.68	0.19
CarolAnn44	0.24	0.0079
Toast42	0.85	0.024

All values represent the average of three independent EMSA experiments (see **MATERIALS AND METHODS** for details on Kd measurement and calculation). The oligonucleotide substrates used in each experiment are described in the respective figure legends with the sequences shown in **Supplementary Table S2**. 'ND' signifies 'not determined' due to incomplete saturation within the data acquisition range.

Supplementary Table S4. *E. coli* K12 strains used for exclusion and papillation assays.

Strain	Relevant Genotype	Reference/construction
LT351	MG1655	From B. Bochner
Cc3LT732	MG1655 *lacI*^{o}<>*kan-Ter*<>*luc*<>*’N pLoL rexB rexA* cI857 *pRoR cro’*<>*lacZYA*$^{+}$	Thomason et al., (2019) (1)
LT772	MG1655 *lacI*^{o}<>*kan-Ter*<>*luc*<>*’N pLoL (rexB rexA)*<>*cat* cI857 *pRoR cro’*<>*lacZYA*$^{+}$	This work
LT1055	MG1655 *ΔlacI-kan luc-N pLoL rexB*$^{+}$ *rexA*$^{+}$ cI857*ind1 pRoR cro27 cII-lacZYA*$^{+}$	Thomason et al., (2021) (2)
LT1886	MG1655 *ΔlacI-kan luc-N pLoL rexB*$^{+}$ *rexA*$^{+}$ cI857 *pRoR cro*$^{+}$ *cII-lacZYA*$^{+}$	Thomason et al. (2021) (2)
LT1892	MG1655 *ΔlacI-kan luc-N pLoL (rexB rexA)*<>*cat* cI857 *pRoR cro*$^{+}$ *cII-lacZYA*$^{+}$	Thomason et al. (2021) (2)
LT2294	MG1655 *ΔlacI-kan luc-N pLoL rexB*$^{+}$ *rexA(R219A/K221A)* cI857 *pRoR cro*$^{+}$ *cII-lacZYA*$^{+}$	This work
LT2298	MG1655 *ΔlacI-kan luc-N pLoL rexB*$^{+}$ *rexA(D215W)* cI857 *pRoR cro*$^{+}$ *cII-lacZYA*$^{+}$	This work
LT2299	MG1655 *ΔlacI-kan luc-N pLoL rexB*$^{+}$ *rexA(D215W)* cI857 *pRoR cro27 cII-lacZYA*$^{+}$	This work
LT2302	MG1655 *ΔlacI-kan luc-N pLoL rexB*$^{+}$ *rexA(Δ239-244)* cI857 *pRoR cro*$^{+}$ *cII-lacZYA*$^{+}$	This work
LT2303	MG1655 *ΔlacI-kan luc-N pLoL rexB*$^{+}$ *rexA(Δ239-244)* cI857 *pRoR cro27 cII-lacZYA*$^{+}$	This work

Supplementary statistical analysis of papillation data

For the data plotted in **Figure 6E**, t-tests were performed with GraphPad Prism software. In all cases, the number of papillae per colony observed with the *rexA* mutants was significantly different from that found with the wildtype RexA strains. For the Cro$^+$ strains, both *rexA* mutants give fewer papillae than do wildtype RexA, while for the *cro27* strains, both *rexA* mutants give more papillae than does the wildtype RexA. When the two *rexA* mutants are compared to each other, in the Cro$^+$ case there is no significant difference between them; however, for the *cro27* case, the two mutants are significantly different with D215W having more papillae/colony in a subset of the colonies. t-test values are as follows:

The difference in papillae/colony between LT1886 Cro$^+$ RexA$^+$ (M = 17.13; SD = 3.59) and LT2302 Cro$^+$ *rexA* Δ239-244 (M = 13.32; SD = 4.42) was significant (t (207) = 6.804; p < <0.0001).

The difference in papillae/colony between LT1886 Cro$^+$ RexA$^+$ (M = 17.13; SD = 3.59) and LT2298 Cro$^+$ *rexA* D215W (M = 14.56; SD = 4.78) was significant (t (202) = 4.332; p < 0.0001).

The difference in papillae/colony between LT1055 *cro27* RexA$^+$ (M = 3.71; SD = 1.80) and LT2303 *cro27 rexA* Δ239-244 (M = 4.613; SD = 2.253) was significant (t (204) = 3.167; p < 0.0018).

The difference in papillae/colony between LT1055 *cro27* RexA$^+$ (M = 3.71; SD = 1.80) and LT2299 *cro27 rexA* D215W (M = 9.234; SD = 7.313) was significant (t (205) = 7.347; p < 0.0001).

The difference in papillae/colony between LT2302 Cro$^+$ *rexA* Δ239-244 (M = 13.32; SD = 4.42) and LT2298 Cro$^+$ *rexA* D215W (M = 14.56; SD = 4.78) was not significant (t (211) = 1.962; p = 0.051).

The difference in papillae/colony between LT2303 *cro27 rexA* Δ239-244 (M = 4.613; SD = 2.253) and LT2299 *cro27 rexA* D215W (M = 9.234; SD = 7.313) was significant (t (211) = 6.219; p < 0.0001).

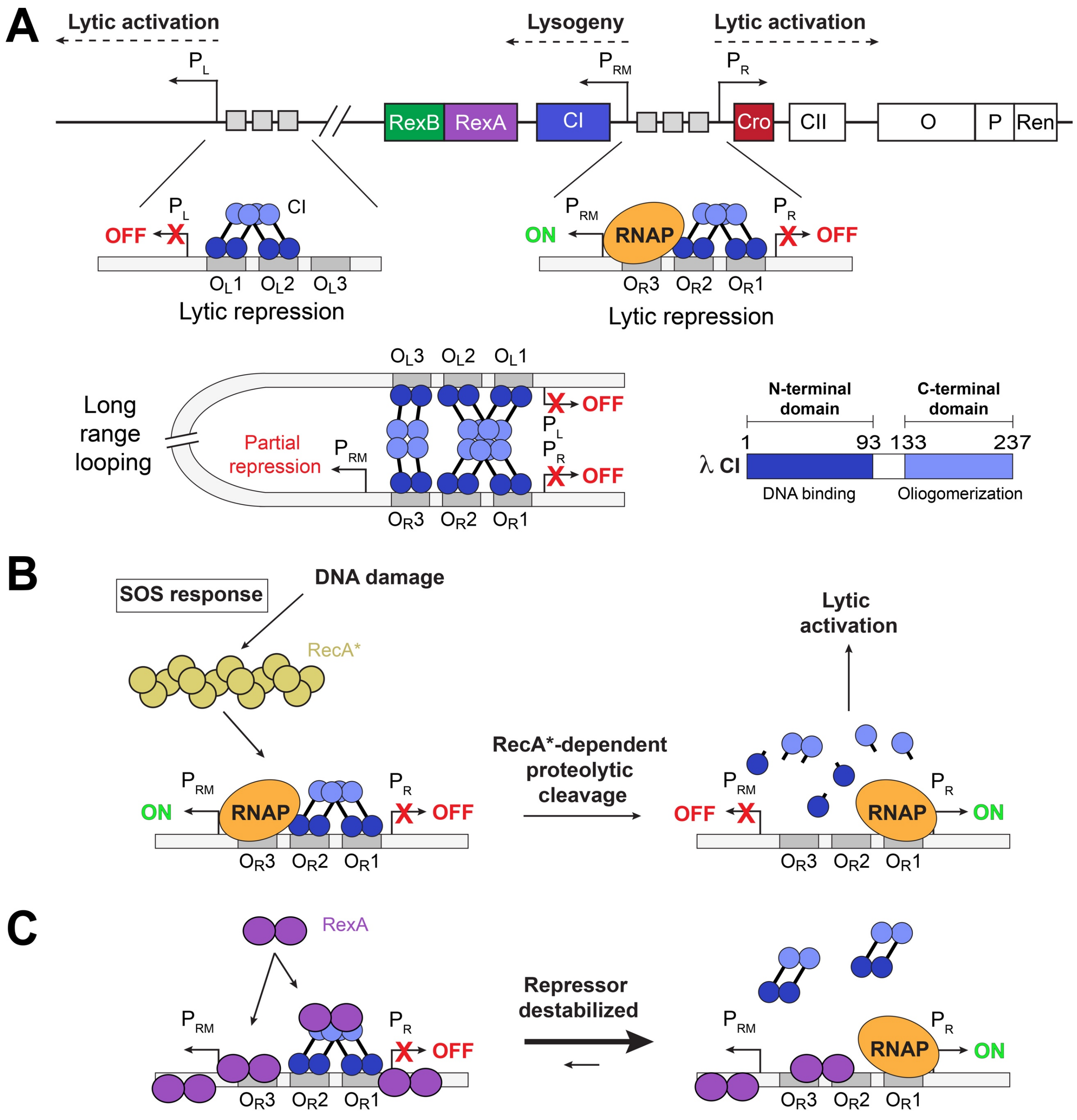

Supplementary Figure S1. Organization and regulation of the λ immunity region. A. Diagram of the λ immunity region. The positions of the P_L, P_{RM} and P_R promoters and neighboring genes are labeled. Associated operator sites are marked by gray boxes. In the lysogenic state, CI dimers (blue) are bound cooperatively to OL1 and OL2 and OR1 and OR2 operators to repress the lytic promotors P_L and P_R, respectively, and direct transcription from the maintenance promoter P_{RM} via RNA polymerase (RNAP, orange) (3, 4). Long-range DNA looping mediated by the further

oligomerization of CI repressor molecules bound to the left and right operators results in stronger repression. Domain organization of CI is shown on the bottom right. B. Prophage induction and lytic activation. Cellular signals like DNA damage and the SOS response activate the RecA protein (RecA*), which promotes proteolytic cleavage of the CI repressor (5). This permits transcription from P_L and P_R lytic promoters. C. Model for RexA modulation of the λ bistable switch. In the absence of RexB, RexA can associate with both the CI repressor (by binding its CTD) and DNA. These protein–protein and protein–DNA interactions may destabilize CI repression and activate the lytic state (2).

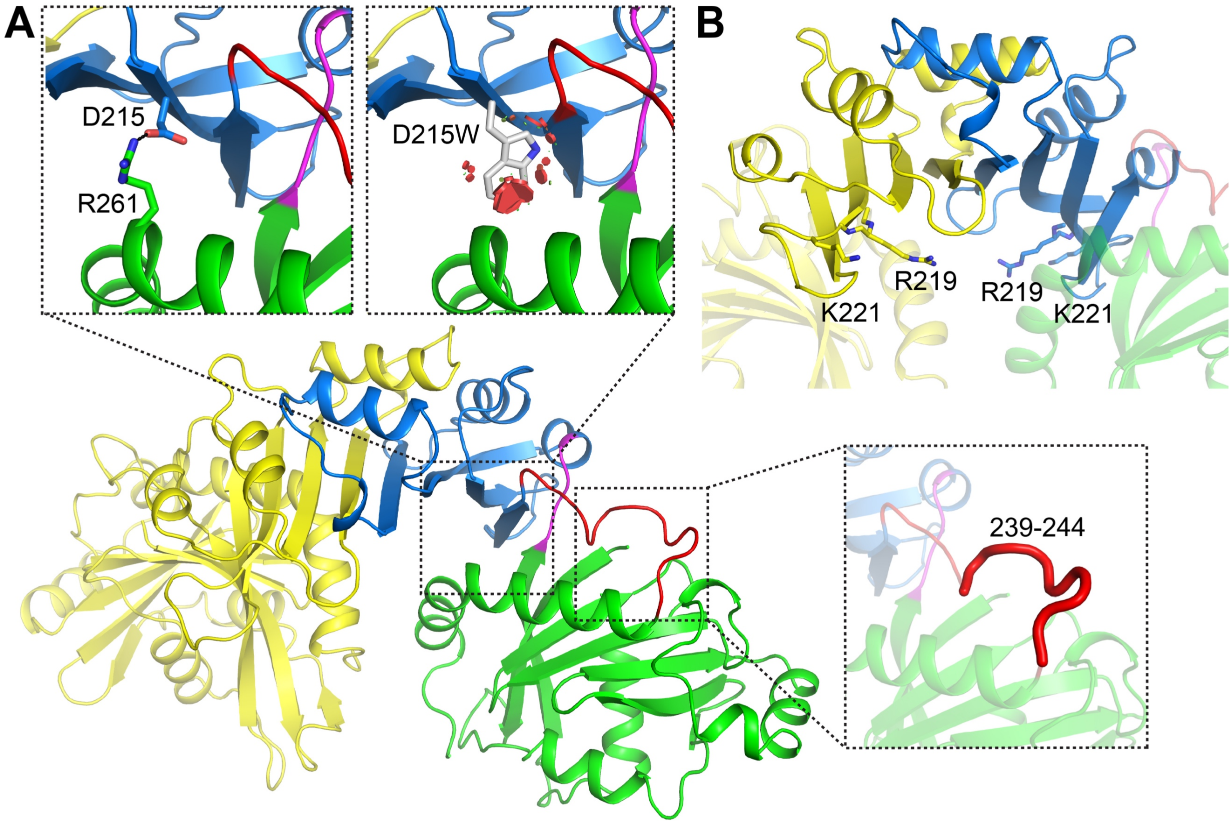

Supplementary Figure S2. Location of RexA mutants. A. Zoomed inserts denote locations of the D215 (blue) and residues 239-244 in the swivel loop (red) that are deleted in the Δ239-244 construct. Modeled D215W mutation (gray) is also shown, with red circles denoting expected steric clashes associated with this substitution. RexA monomers are colored in as in **Figure 1**. B. Location of R219 and K221 side chains in the dimerization domain.

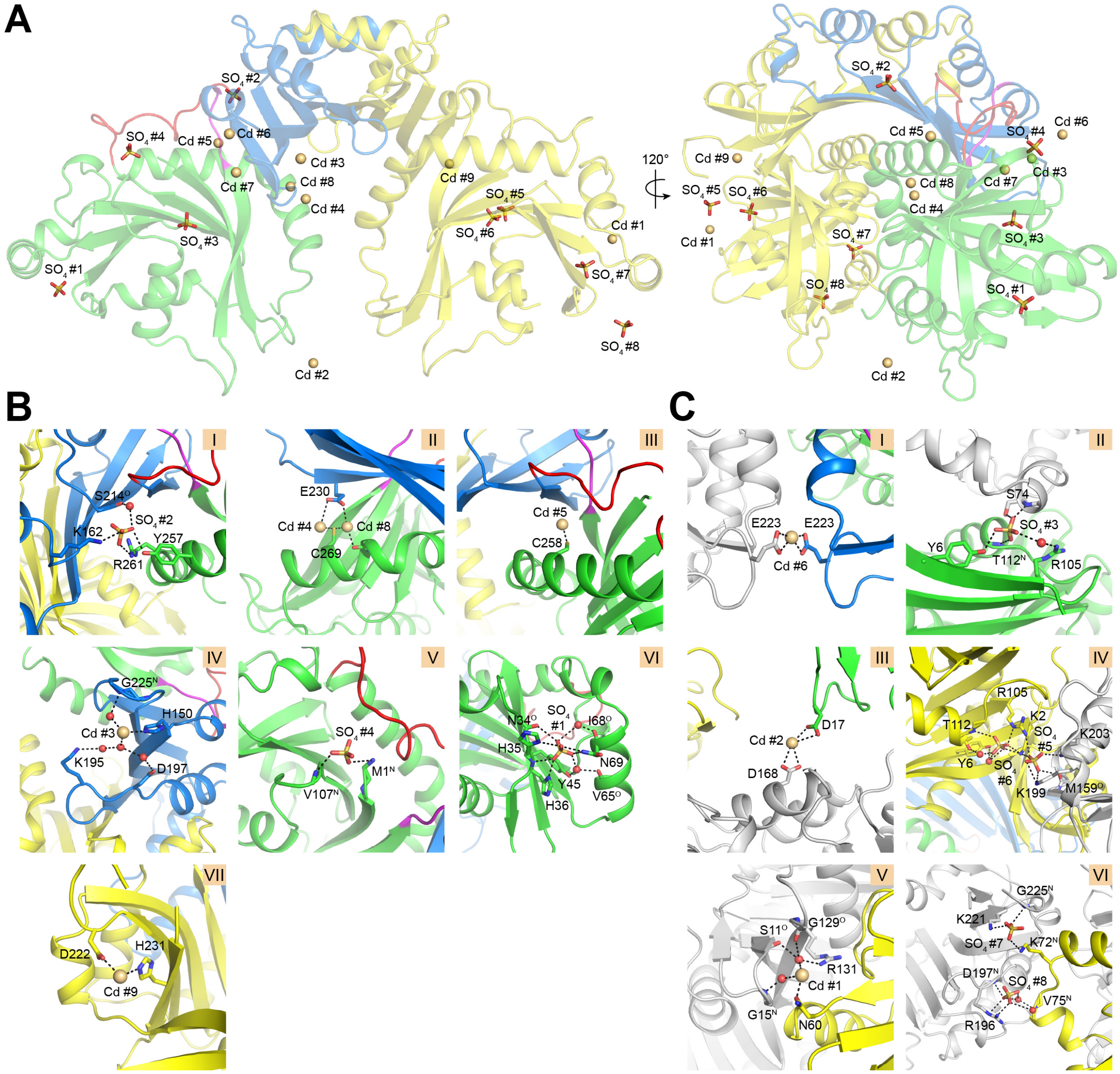

Supplementary Figure S3. **Structural coordination of bound ions and sulfates.** A. Location of ordered sulfates (SO_4) and cadmium (Cd) ions associated with the RexA dimer following crystallization. Ions are numbered for reference (see B and C). RexA dimer is colored as in **Figure 1**. B. Zoomed views intramolecular ionic interactions. Interacting side chains are labeled with hydrogen bonds shown as dashed black lines. Superscripts "N" and "O" denote backbone nitrogen and carbonyl oxygens, respectively. Associated water molecules (red spheres) are shown where applicable. C. Zoomed views of intermolecular ionic interactions mediating crystal contacts. Symmetry-related molecules are colored gray.

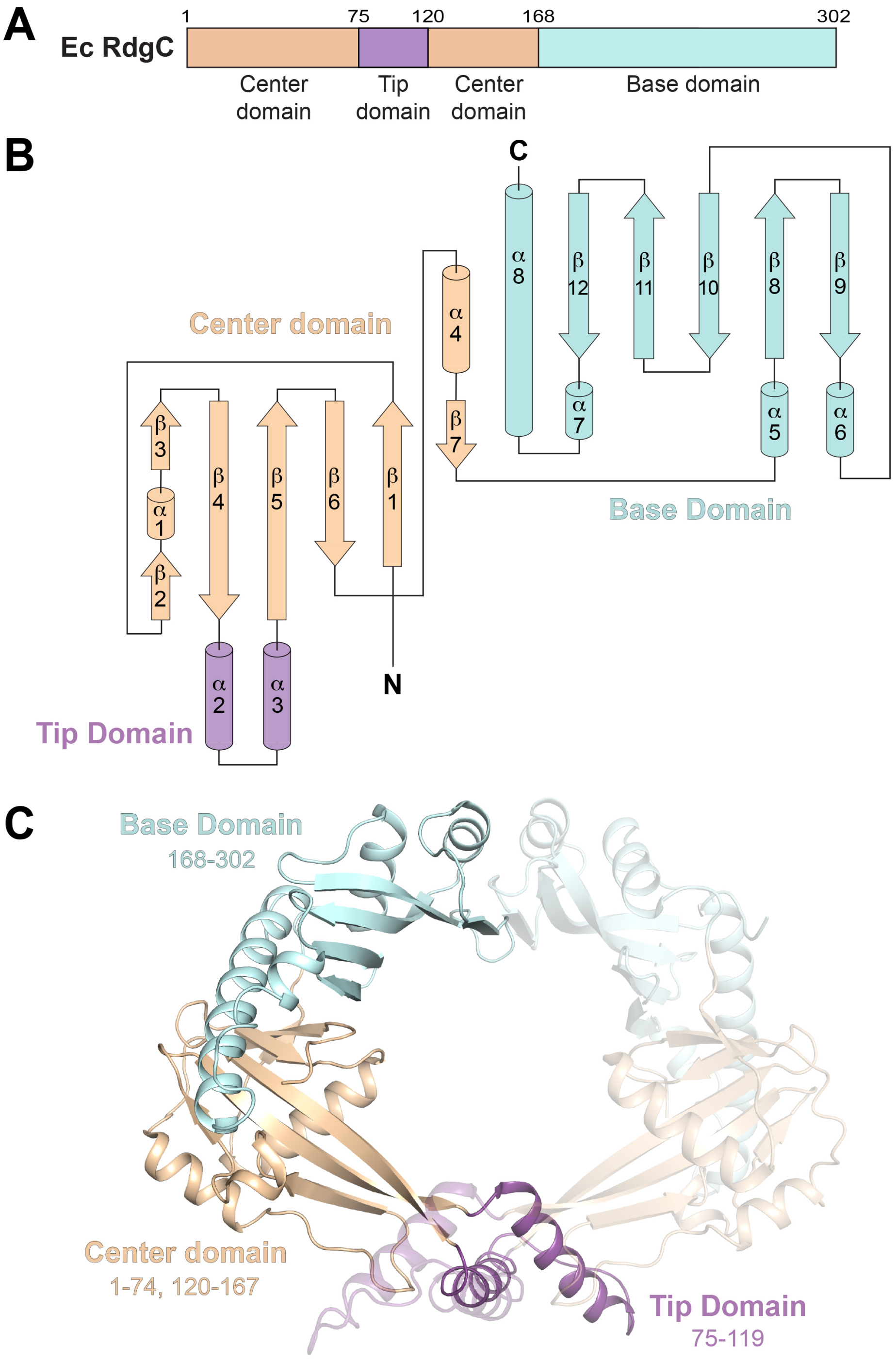

Supplementary Figure S4. Structure and topology of *E. coli* RdgC. A. Domain architecture of *E. coli* (Ec) RdgC. B. Topology diagram of *E. coli* RdgC monomer with coloring as in B. C. Structure of *E. coli* RdgC dimer (PDB: 2OWL). The center, tip, and base domains are colored beige, purple, and cyan respectively in one monomer with accompanying residue numbering. Second monomer is colored the same but rendered partially transparent for contrast.

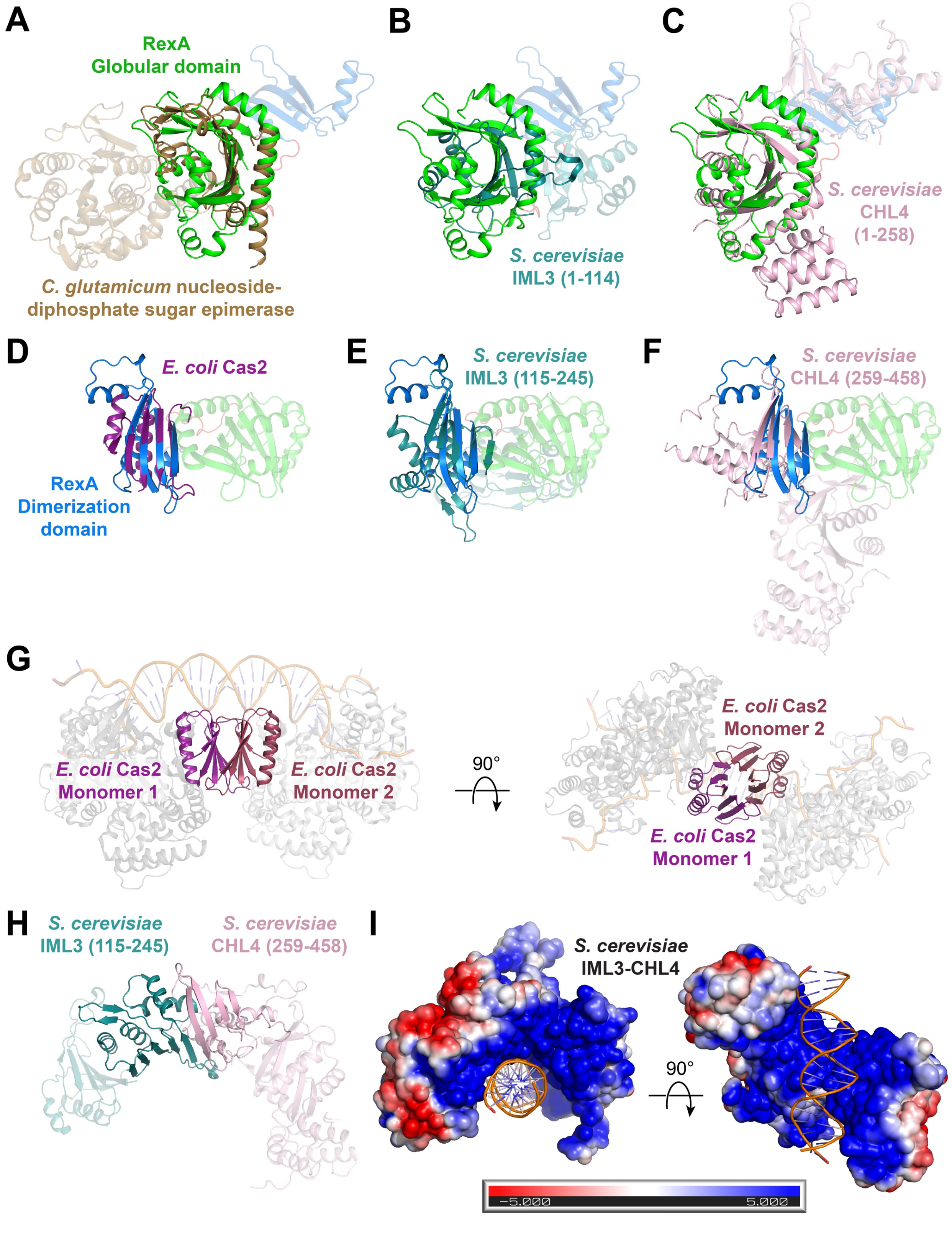
A
RexA
Globular domain
C. glutamicum nucleoside-
diphosphate sugar epimerase
B
S. cerevisiae
IML3 (1-114)
C
S. cerevisiae
CHL4
(1-258)
D
E. coli Cas2
RexA
Dimerization
domain
E
S. cerevisiae
IML3 (115-245)
F
S. cerevisiae
CHL4 (259-458)
G
E. coli Cas2
Monomer 1
E. coli Cas2
Monomer 2
90°
E. coli Cas2
Monomer 2
E. coli Cas2
Monomer 1
H
S. cerevisiae
IML3 (115-245)
S. cerevisiae
CHL4 (259-458)
I
S. cerevisiae
IML3-CHL4
90°
−5.000
5.000

Supplementary Figure S5. **Additional structural homologies identified by Dali.** A-F, Superposition of λ RexA with a nucleoside-diphosphate sugar epimerase from *Corynebacterium glutamicum* (olive, PDB: 3OH8, Z-score 4.1, RMSD 4.4 Å), *Saccharomyces cerevisiae* IML3 (teal) and CHL4 (pink) (PDB: 8OVW, Z-score 4.1, RMSD 5.1 Å), and *E. coli* Cas2 (purple, PDB: 5DLJ, Z-score 4, RMSD 3 Å) illustrating structural homologies that are shared with RexA's globular (green) and dimerization (blue) domains, respectively. G. Orientation of Cas2 dimerization within Cas1-Cas2 dual-forked DNA complexes (PDB: 5DLJ) involved in CRISPR spacer acquisition. Cas 2 monomers are colored purple and ruby, respectively. H. The C-terminal segments of IML3 (teal) and CHL4 (pink) heterodimerize within the centromere-associated inner kinetochore (CCAN) complex (PDB: 8OVW) in a manner that creates an extend anti-parallel β-sheet across the dimer interface. I. Electrostatic surface of *Saccharomyces cerevisiae* IML3-CHL4 heterodimer. Scale bar indicates electrostatic surface coloring from -5 K_bT/e_c to +5 K_bT/e_c. Centromeric DNA from the bound CANN complex (PDB: OVW) is shown.

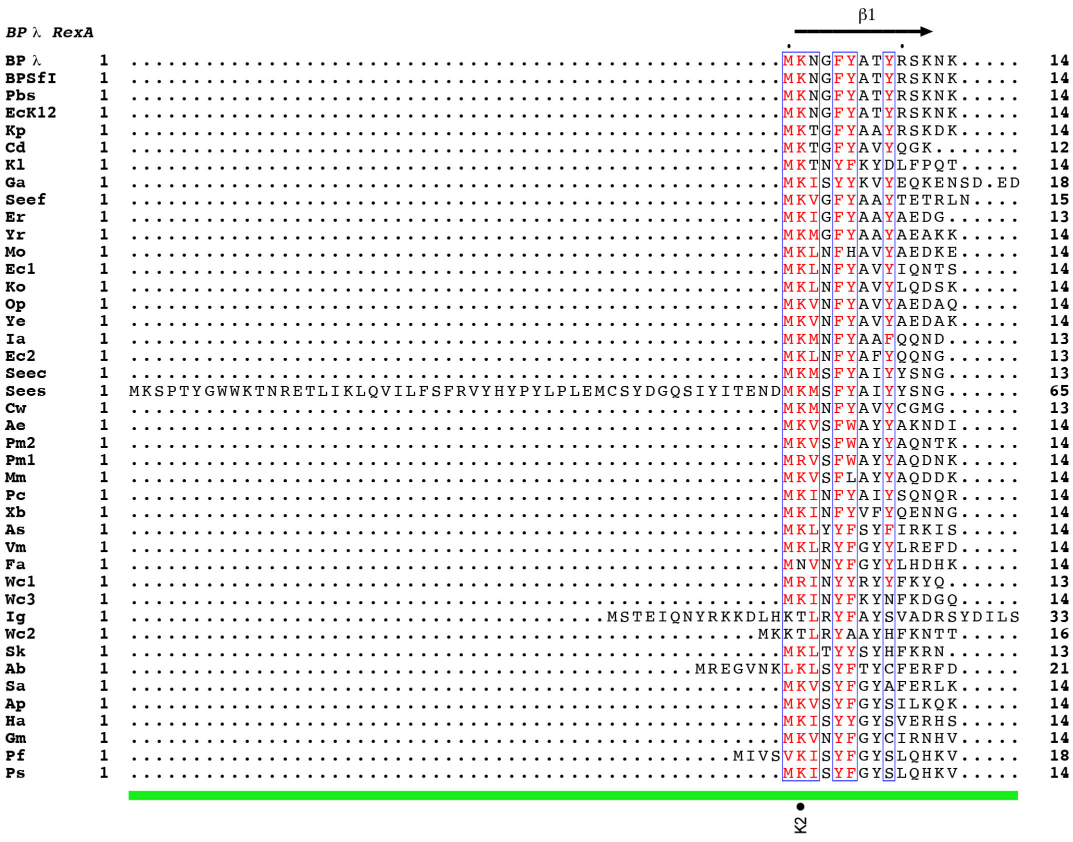
β1
BP λ RexA
BP λ 1 ....MKNGFYATYRSKNK..... 14
BPSfI 1 ....MKNGFYATYRSKNK..... 14
Pbs 1 ....MKNGFYATYRSKNK..... 14
EcK12 1 ....MKNGFYATYRSKNK..... 14
Kp 1 ....MKTGFYAAYRSKDK..... 14
Cd 1 ....MKTGFYAVYQGK....... 12
Kl 1 ....MKTNYFKYDLFPQT..... 14
Ga 1 ....MKISYYKVYEQKENSD.ED 18
Seef 1 ....MKVGFYAAYTETRLN.... 15
Er 1 ....MKIGFYAAYAEDG...... 13
Yr 1 ....MKMGFYAAYAEAKK..... 14
Mo 1 ....MKLNFHAVYAEDKE..... 14
Ec1 1 ....MKLNFYAVYIQNTS..... 14
Ko 1 ....MKLNFYAVYLQDSK..... 14
Op 1 ....MKVNFYAVYAEDAQ..... 14
Ye 1 ....MKVNFYAVYAEDAK..... 14
Ia 1 ....MKMNFYAAFQQND...... 13
Ec2 1 ....MKLNFYAFYQQNG...... 13
Seec 1 ....MKMSFYAIYYSNG...... 13
Sees 1 MKSPTYGWWKTNRETLIKLQVILFSFRVYHYPYLPLEMCSYDGQSIYITENDMKMSFYAIYYSNG...... 65
Cw 1 ....MKMNFYAVYCGMG...... 13
Ae 1 ....MKVSFWAYYAKNDI..... 14
Pm2 1 ....MKVSFWAYYAQNTK..... 14
Pm1 1 ....MRVSFWAYYAQDNK..... 14
Mm 1 ....MKVSFLAYYAQDDK..... 14
Pc 1 ....MKINFYAIYSQNQR..... 14
Xb 1 ....MKINFYVFYQENNG..... 14
As 1 ....MKLYYFSYFIRKIS..... 14
Vm 1 ....MKLRYFGYYLREFD..... 14
Fa 1 ....MNVNYFGYYLHDHK..... 14
Wc1 1 ....MRINYYRYYFKYQ...... 13
Wc3 1 ....MKINYFKYNFKDGQ..... 14
Ig 1 ....MSTEIQNYRKKDLHKTLRYFAYSVADRSYDILS 33
Wc2 1 ....MKKTLRYAAYHFKNTT..... 16
Sk 1 ....MKLTYYSYHFKRN...... 13
Ab 1 ....MREGVNKLKLSYFTYCFERFD..... 21
Sa 1 ....MKVSYFGYAFERLK..... 14
Ap 1 ....MKVSYFGYSILKQK..... 14
Ha 1 ....MKISYYGYSVERHS..... 14
Gm 1 ....MKVNYFGYCIRNHV..... 14
Pf 1 ....MIVSVKISYFGYSLQHKV..... 18
Ps 1 ....MKISYFGYSLQHKV..... 14
K2

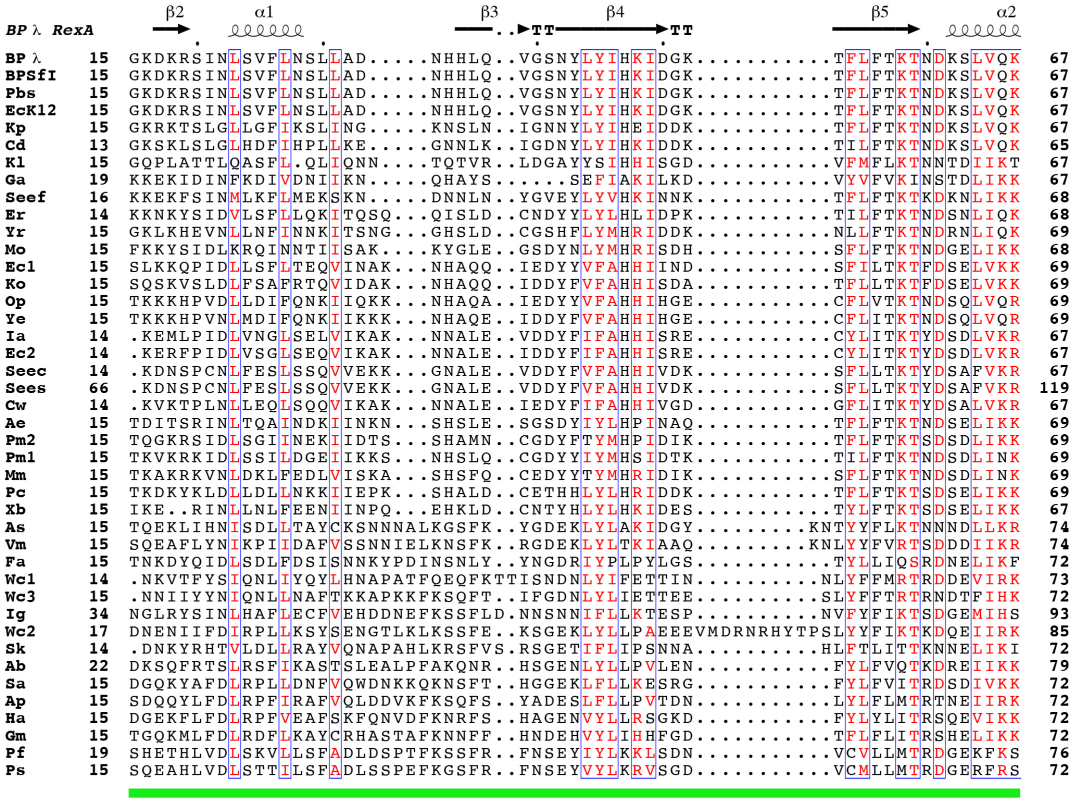
β2 α1 β3 β4 β5 α2
TT TT
BP λ RexA
BP λ 15 GKDKRSINLSVFLNSLLAD.....NHHLQ..VGSNYLYIHKIDGK..........TFLFTKTNDKSLVQK 67
BPSfI 15 GKDKRSINLSVFLNSLLAD.....NHHLQ..VGSNYLYIHKIDGK..........TFLFTKTNDKSLVQK 67
Pbs 15 GKDKRSINLSVFLNSLLAD.....NHHLQ..VGSNYLYIHKIDGK..........TFLFTKTNDKSLVQK 67
EcK12 15 GKDKRSINLSVFLNSLLAD.....NHHLQ..VGSNYLYIHKIDGK..........TFLFTKTNDKSLVQK 67
Kp 15 GKRKTSLGLLGFIKSLING.....KNSLN..IGNNYLYIHEIDDK..........TFLFTKTNDKSLVQK 67
Cd 13 GKSKLSLGLHDFIHPLLKE.....GNNLK..IGDNYLYIHKIDDK..........TILFTKTNDKSLVQK 65
Kl 15 GQPLATTLQASFL.QLIQNN....TQTVR..LDGAYYSIHHISGD..........VFMFLKTNNTDIIKT 67
Ga 19 KKEKIDINFKDIVDNIIKN.....QHAYS......SEFIAKILKD..........VYVFVKINSTDLIKK 67
Seef 16 KKEKFSINMLKFLMEKSKN.....DNNLN..YGVEYLYVHKINNK..........TFLFTKTKDKNLIKK 68
Er 14 KKNKYSIDVLSFLLQKITQSQ...QISLD..CNDYYLYLHLIDPK..........TILFTKTNDSNLIQK 68
Yr 15 GKLKHEVNLLNFINNKITSNG...GHSLD..CGSHFLYMHRIDDK..........NLLFTKTNDRNLIQK 69
Mo 15 FKKYSIDLKRQINNTIISAK....KYGLE..GSDYNLYMHRISDH..........SFLFTKTNDGELIKK 68
Ec1 15 SLKKQPIDLLSFLTEQVINAK...NHAQQ..IEDYYVFAHHIIND..........SFILTKTFDSELVKK 69
Ko 15 SQSKVSLDLFSAFRTQVIDAK...NHAQQ..IDDYFVFAHHISDA..........TFLLTKTFDSELVKK 69
Op 15 TKKKHPVDLLDIFQNKIIQKK...NHAQA..IEDYYVFAHHIHGE..........CFLVTKTNDSQLVQR 69
Ye 15 TKKKHPVNLMDIFQNKIIKKK...NHAQE..IDDYFVFAHHIHGE..........CFLITKTNDSQLVQR 69
Ia 14 .KEMLPIDLVNGLSELVIKAK...NNALE..VDDYFIFAHHISRE..........CYLITKTYDSDLVKR 67
Ec2 14 .KERFPIDLVSGLSEQVIKAK...NNALE..IDDYFIFAHHISRE..........CYLITKTYDSDLVKR 67
Seec 14 .KDNSPCNLFESLSSQVVEKK...GNALE..VDDYFVFAHHIVDK..........SFLLTKTYDSAFVKR 67
Sees 66 .KDNSPCNLFESLSSQVVEKK...GNALE..VDDYFVFAHHIVDK..........SFLLTKTYDSAFVKR 119
Cw 14 .KVKTPLNLLEQLSQQVIKAK...NNALE..IEDYFIFAHHIVGD..........GFLITKTYDSALVKR 67
Ae 15 TDITSRINLTQAINDKIINKN...SHSLE..SGSDYIYLHPINAQ..........TFLFTKTNDSELIKK 69
Pm2 15 TQGKRSIDLSGIINEKIIDTS...SHAMN..CGDYFTYMHPIDIK..........TFLFTKTSDSDLIKK 69
Pm1 15 TKVRKKIDLSSILDGEIIKKS...NHSLQ..CGDYYIYMHSIDTK..........TILFTKTNDSDLINK 69
Mm 15 TKAKRKVNLDKLFEDLVISKA...SHSFQ..CEDYYTYMHRIDIK..........SFLFTKTNDSDLINK 69
Pc 15 TKDKYKLDLLDLLNKKIIEPK...SHALD..CETHHLYLHRIDDK..........TFLFTKTSDSELIKK 69
Xb 15 IKE..RINLLNLFEENIINPQ...EHKLD..CNTYHLYLHKIDES..........TYLFTKTSDSELIKK 67
As 15 TQEKLIHNISDLLTAYCKSNNNALKGSFK..YGDEKLYLAKIDGY.........KNTYYFLKTNNNDLLKR 74
Vm 15 SQEAFLYNIKPIIDAFVSSNNIELKNSFK..RGDEKLYLTKIAAQ.........KNLYYFVRTSDDDIIKR 74
Fa 15 TNKDYQIDLSDLFDSISNNKYPDINSNLY..YNGDRIYPLPYLGS..........TYLLIQSRDNELIKF 72
Wc1 14 .NKVTFYSIQNLIYQYLHNAPATFQEQFKTTISNDNLYIFETTIN.........NLYFFMRTRDDEVIRK 73
Wc3 15 .NNIIYYNLQNLLNAFTKKAPKKFKSQFT..IFGDNLYLIETTEE.........SLYFFTRTRNDTFIHK 72
Ig 34 NGLRYSINLHAFLECFVEHDDNEFKSSFLD.NNSNNIFLLKTESP.........NVFYFIKTSDGEMIHS 93
Wc2 17 DNENIIFDIRPLLKSYSENGTLKLKSSFE..KSGEKLYLLPAEEEVMDRNRHYTPSLYYFIKTKDQEIIRK 85
Sk 14 .DNKYRHTVLDLLRAYVQNAPAHLKRSFVS.RSGETIFLIPSNNA.........HLFTLITTKNNELIKI 72
Ab 22 DKSQFRTSLRSFIKASTSLEALPFAKQNR..HSGENLYLLPVLEN..........FYLFVQTKDREIIKK 79
Sa 15 DGQKYAFDLRPLLDNFVQWDNKKQKNSFT..HGGEKLFLLKESRG..........FYLFVITRDSDIVKK 72
Ap 15 SDQQYLFDLRPFIRAFVQLDDVKFKSQFS..YADESLFLLPVTDN..........LYLFLMTRTNEIIRK 72
Ha 15 DGEKFLFDLRPFVEAFSKFQNVDFKNRFS..HAGENVYLLRSGKD..........FYLYLITRSQEVIKK 72
Gm 15 TGQKMLFDLRDFLKAYCRHASTAFKNNFF..HNDEHVYLIHHFGD..........TFLFLITRSHELIKK 72
Pf 19 SHETHLVDLSKVLLSFADLDSPTFKSSFR..FNSEYIYLKKLSDN..........VCVLLMTRDGEKFKS 76
Ps 15 SQEAHLVDLSTTILSFADLSSPEFKGSFR..FNSEYVYLKRVSGD..........VCMLLMTRDGERFRS 72

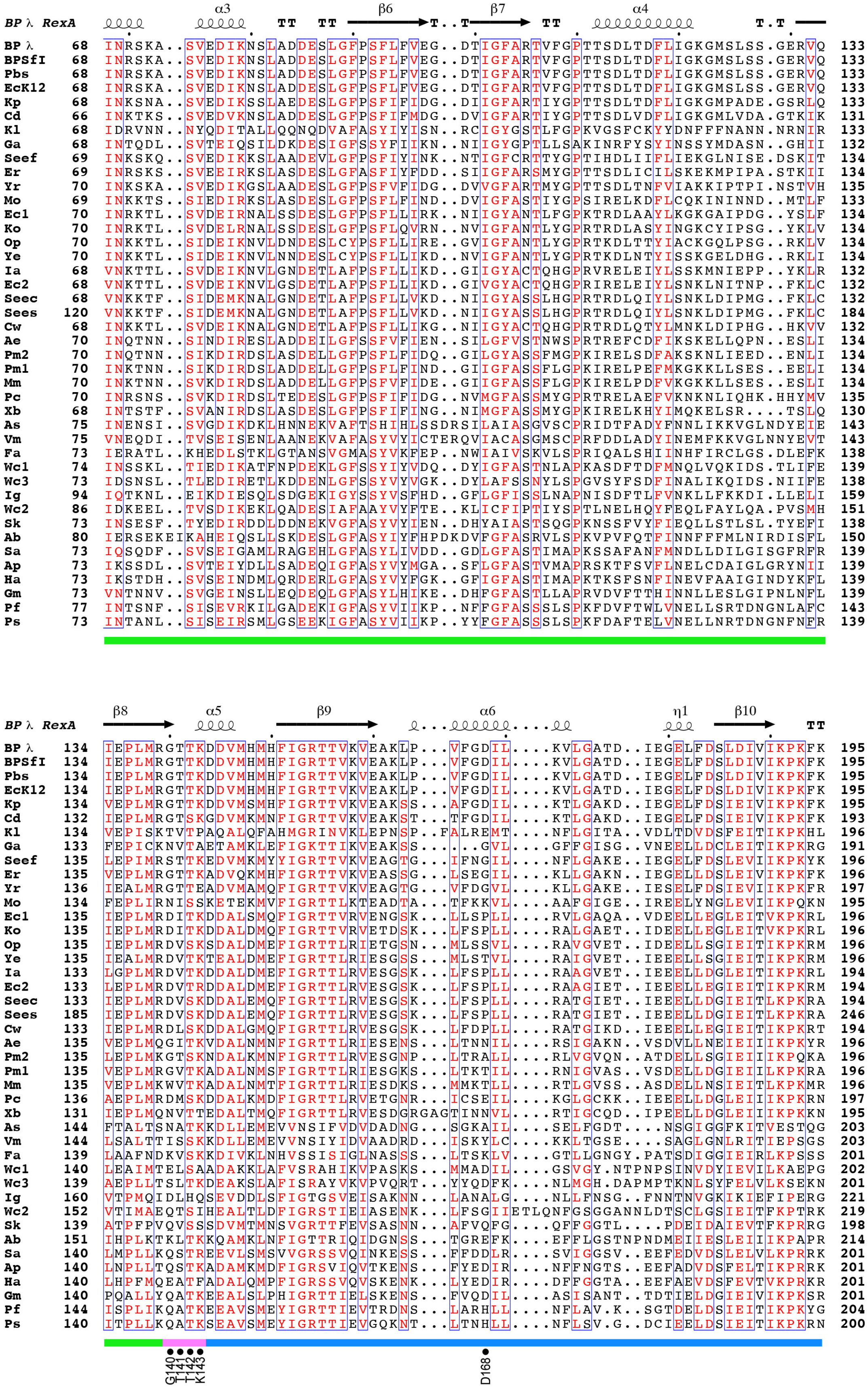

BP λ RexA
α3 TT TT β6 T..T β7 TT α4 T.T
BP λ 68 INRSKA..SVEDIKNSLADDESLGFPSFLFVEG..DTIGFARTVFGPTTSDLTDFLIGKGMSLSS.GERVQ 133
BPSfI 68 INRSKA..SVEDIKNSLADDESLGFPSFLFVEG..DTIGFARTVFGPTTSDLTDFLIGKGMSLSS.GERVQ 133
Pbs 68 INRSKA..SVEDIKNSLADDESLGFPSFLFVEG..DTIGFARTVFGPTTSDLTDFLIGKGMSLSS.GERVQ 133
EcK12 68 INRSKA..SVEDIKNSLADDESLGFPSFLFVEG..DTIGFARTVFGPTTSDLTDFLIGKGMSLSS.GERVQ 133
Kp 68 INKSNA..SVEDIKSSLAEDESLGFPSFIFIDG..DIIGFARTIYGPTTSDLTDFLIGKGMPADE.GSRLQ 133
Cd 66 INKTKS..SVEDVKNSLADDESLGFPSFIFMDG..DVIGFARTIYGPTTSDLVDFLIGKGMLVDA.GTKIK 131
Kl 68 IDRVNN..NYQDITALLQQNQDVAFASYIYISN..RCIGYGSTLFGPKVGSFCKYYDNFFFNANN.NRNIR 133
Ga 68 INTQDL..SVTEIQSILDKDESIGFSSYFIIKN..NIIGYGPTLLSAKINRFYSYINSSYMDASN..GHII 132
Seef 69 INKSKQ..SVEDIKSLAADEVLGFPSFIYINK..NTIGFCRTTYGPTIHDLIIFLIEKGLNISE.DSKIT 134
Er 69 INRSKS..SVEEIRKSLASDESLGFASFIYFDD..SIIGFARSMYGPTTSDLICILSKEKMPIPA.STKII 134
Yr 70 INKSKA..SVEDIKGSLAADESLGFPSFVFIDG..DVVGFARTMYGPTTSDLTNFVIAKKIPTPI.NSTVH 135
Mo 69 INKKTS..SIEDIRKSLASDESLGFPSFLFIND..DVIGFASTIYGPSIRELKDFLCQKININND..MTLF 133
Ec1 70 INRKTL..SVDEIRNALSSDESLGFPSFLLIRK..NIIGYANTLFGPKTRDLAAYLKGKGAIPDG..YSLF 134
Ko 70 INRKTL..SVDELRNALSSDESLGFPSFLQVRN..NVIGFANTLYGPRTRDLASYINGKCYIPSG..YKLV 134
Op 70 INKKTL..SIDEIKNVLDNDESLCYPSFLLIRE..GVIGFANTLYGPRTKDLTTYIACKGQLPSG..RKLV 134
Ye 70 INKKTL..SIDEIKNVLNNDESLCYPSFLLIKD..NVIGFANTLYGPRTKDLNTYISSKGELDHG..RKLI 134
Ia 68 VNKTTL..SVDEIKNVLGNDETLAFPSFLLIKD..GIIGYACTQHGPRVRELEIYLSSKMNIEPP..YKLR 132
Ec2 68 VNKTTL..SVDEIKNVLGSDETLAFPSFLLIKD..GIVGYACTQHGPRIRELEIYLSNKLNITNP..FKLC 132
Seec 68 VNKKTF..SIDEMKNALGNDETLAFPSFLLVKD..NIIGYASSLHGPRTRDLQIYLSNKLDIPMG..FKLC 132
Sees 120 VNKKTF..SIDEMKNALGNDETLAFPSFLLVKD..NIIGYASSLHGPRTRDLQIYLSNKLDIPMG..FKLC 184
Cw 68 INKKTL..SVDEIKNALGNDETLAFPSFLLIKG..NIIGYACTQHGPRTRDLQTYLMNKLDIPHG..HKVV 132
Ae 70 INQTNN..SINDIRESLADDEILGFSSFVFIEN..SILGFVSTNWSPRTREFCDFIKSKELLQPN..ESLI 134
Pm2 70 INQTNN..SIKDIRDSLASDELLGFPSFLFIDQ..GILGYASSFMGPKIRELSDFAKSKNLIEED..ENLI 134
Pm1 70 INKTNN..SIKDIRDSLADDELLGFPSFLFIND..GIIGFASSFLGPKIRELPEFMKGKKLLSES..EELI 134
Mm 70 INKTNN..SVKDIRDSLADDELLGFPSFVFIDE..GIIGFASSFLGPKIRELPDFVKGKKLLSES..ESLI 134
Pc 70 INRSNS..SVKDIRDSLTEDESLGFPSFIFIDG..NVMGFASSMYGPRTRELAEFVKNKNLIQHK.HHYMV 135
Xb 68 INTSTF..SVANIRDSLASDESLGFPSFIFING..NIMGFASSMYGPKIRELKHYIMQKELSR....TSLQ 130
As 75 INENSI..SVGDIKDKLHNNEKVAFTSHIHLSSDRSILAIASGVSCPRIDTFADYFNNLIKKVGLNDYEIE 143
Vm 75 VNEQDI..TVSEISENLAANEKVAFASYVYICTERQVIACASGMSCPRFDDLADYINEMFKKVGLNNYEVT 143
Fa 73 IERATL..KHEDLSTKLGTANSVGMASYVKFEP..NWIAIVSKVLSPRIQALSHIINHFIRCLGS.DLEFK 138
Wc1 74 INSSKL..TIEDIKATFNPDEKLGFSSYIYVDQ..DYIGFASTNLAPKASDFTDFMNQLVQKIDS.TLIFE 139
Wc3 73 IDSNSL..TLEDIRETLKDDENLGFSSYVYVGK..DYLAFSSNYLSPGVSYFSDFINALIKQIDS.NIIFE 138
Ig 94 IQTKNL..EIKDIESQLSDGEKIGYSSYVSFHD..GFLGFISSLNAPNISDFTLFVNKLLFKKDI.LLELI 159
Wc2 86 IDKEEL..TVSDIKEKLQADESIAFAAYVYFTE..KLICFIPTIYSPTLNELHQYFEQLFAYLQA.PVSMH 151
Sk 73 INSESF..TYEDIRDDLDDNEKVGFASYVYIEN..DHYAIASTSQGPKNSSFVYFIEQLLSTLSL.TYEFI 138
Ab 80 IERSEKEIKAHEIQSLLSKDESLGFASYIYFHPDKDVFGFASRVLSPKVPVFQTFINNFFFMLNIRDISFL 150
Sa 73 IQSQDF..SVSEIGAMLRAGEHLGFASYLIVDD..GDLGFASTIMAPKSSAFANFMNDLLDILGISGFRFR 139
Ap 73 IKSSDL..SVTEIYDLLSADEQIGFASYVYMGA..SFLGFASTVMAPRSKTFSVFLNELCDAIGLGRYNII 139
Ha 73 IKSTDH..SVSEINDMLQRDERLGFASYVYFGK..GFIGFASTIMAPKTKSFSNFINEVFAAIGINDYKFL 139
Gm 73 VNTNNV..SVGEINSLLEQDEQLGFASYLHIKE..DHFGFASTLLAPRVDVFTTHINNLLESLGIPNLNFL 139
Pf 77 INTSNF..SISEVRKILGADEKIGFASYVIIKP..NFFGFASSSLSPKFDVFTWLVNELLSRTDNGNLAFC 143
Ps 73 INTANL..SISEIRSMLGSEEKIGFASYVIIKP..YYFGFASSSLSPKFDAFTELVNELLNRTDNGNFNFR 139
BP λ RexA
β8 α5 β9 α6 η1 β10 TT
BP λ 134 IEPLMRGTTKDDVMHMHFIGRTTVKVEAKLP...VFGDIL....KVLGATD..IEGELFDSLDIVIKPKFK 195
BPSfI 134 IEPLMRGTTKDDVMHMHFIGRTTVKVEAKLP...VFGDIL....KVLGATD..IEGELFDSLDIVIKPKFK 195
Pbs 134 IEPLMRGTTKDDVMHMHFIGRTTVKVEAKLP...VFGDIL....KVLGATD..IEGELFDSLDIVIKPKFK 195
EcK12 134 IEPLMRGTTKDDVMHMHFIGRTTVKVEAKLP...VFGDIL....KVLGATD..IEGELFDSLDIVIKPKFK 195
Kp 134 VEPLMRGTTKDDVMSMHFIGRTTVKVEAKSS...AFGDIL....KTLGAKD..IEGELFDSIEIVIKPKFK 195
Cd 132 IEPLMRGTSKGDVMKMNFIGRTTVKVEAKST...TFGDIL....KTLGAKD..IEGELFDSIEIVIKPKFK 193
Kl 134 VEPISKTVTPAQALQFAHMGRINVKLEPNSP..FALREMT....NFLGITA..VDLTDVDSFEITIKPKHL 196
Ga 133 FEPICKNVTAETAMKLEFIGKTTIKVEAKSS......GVL....GFFGISG..VNEELLDCLEITIKPKRG 191
Seef 135 LEPIMRSTTKEDVMKMYYIGRTTVKVEAGTG...IFNGIL....NFLGAKE..IEGELFDSLEVIIKPKYK 196
Er 135 VEPLMRGTTKADVQKMHFIGRTTVKVEASSG...LSEGIL....KLLGAKN..IEGELLDSLEIVIKPKFK 196
Yr 136 IEALMRGTTEADVMAMQFIGRTTVKVEAGTG...VFDGVL....KLLGAKE..IESELFDSIEVIIKPKFR 197
Mo 134 FEPLIRNISSKETEKMVFIGRTTLKTEADTA...TFKKVL....AAFGIGE..IREELYNGLEVIIKPQKN 195
Ec1 135 IEPLMRDITKDDALSMQFIGRTTVRVENGSK...LLSPLL....RVLGAQA..VDEELLEGLEITVKPKRL 196
Ko 135 IEPLMRDITKDDALDMQFIGRTTVRVETDSK...LFSPLL....RALGAET..IDEELLEGLEITVKPKRL 196
Op 135 IEPLMRDVSKSDALDMEFIGRTTLRIETGSN...MLSSVL....RAVGVET..IDEELLSGIEITIKPKRM 196
Ye 135 IEALMRDVTKTEALDMEFIGRTTLRIESGSS...MLSTVL....RAIGVET..IEEELLSGIEITIKPKRM 196
Ia 133 LGPLMRDVTKDDALDMEFIGRTTLRVESGSK...LFSPLL....RAAGVET..IEEELLDGLEITIKPKRL 194
Ec2 133 IEPLMRDVTRDDALDMEFIGRTTLRVESGSK...LFSPLL....RAAGIET..IEEELLEGIEITIKPKRM 194
Seec 133 IEPLMRDVSKDDALEMQFIGRTTLRVESGSK...LFSPLL....RATGIET..IEEELLDGIEITLKPKRA 194
Sees 185 IEPLMRDVSKDDALEMQFIGRTTLRVESGSK...LFSPLL....RATGIET..IEEELLDGIEITLKPKRA 246
Cw 133 IEPLMRDLSKDDALGMQFIGRTTLRVESGSK...LFDPLL....RATGIKD..IEEELLEGIEITIKPKRT 194
Ae 135 VEPLMQGITKVDALNMNFIGRTTLRIESENS...LTNNIL....RSIGAKN..VSDVLLNEIEIIIKPKYR 196
Pm2 135 LEPLMKGTSKNDALKMNFIGRTTLRVESGNP...LTRALL....RLVGVQN..ATDELLSGIEIIIKPQKA 196
Pm1 135 VEPLMRGVTKADALNMSFIGRTTLRIESGKS...LTKTIL....RNIGVAS..VSDELLDGIEIILKPKRA 196
Mm 135 VEPLMKWVTKADALNMTFIGRTTLRIESDKS...MMKTLL....RTLGVSS..ASDELLNSIEITLKPKMR 196
Pc 136 AEPLMRDMSKDDALKMDFIGRTTLRVESGKR...ICSEIL....KGLGCKK..IEEELLDGLEIIIKPKRN 197
Xb 131 IEPLMQNVTTEDALTMQFIGRTTLRVESDGRGAGTINNVL....RTIGCQD..IPEELLDGLEIIIKPKKN 195
As 144 FTALTSNATKKDLLEMEVVNSIFVDVDADNG...SGKAIL....SELFGDT....NSGIGGFKITVESTQG 203
Vm 144 LSALTTISSKKDLLEMEVVNSIYIDVDAARD...ISKYLC....KKLTGSE....SAGLGNLRITIEPSGS 203
Fa 139 LAAFNDKVSKKDIVKLNHVSSISIGLNASSS...LTSKLV....GTLLGNGY.PATSDIGGIEIRLKPSSS 201
Wc1 140 LEAIMTELSAADAKKLAFVSRAHIKVPASKS...MMADIL....GSVGY.NTPNPSINVDYIEVILKAEPG 202
Wc3 139 AEPLLTSLTKDEAKSLAFISRAYVKVPVQRT...YYQDFK....NLMGH.DAPMPTKNLSYFELVLKSEKN 201
Ig 160 VTPMQIDLHQSEVDDLSFIGTGSVEISAKNN...LANALG....NLLFNSG..FNNTNVGKIKIEFIPERG 221
Wc2 152 VTIMAEQTSIHEALTLDFIGRSTIEIASENK...LFSGIIETLQNFGSGGANNLDTSCLGSIEITFKPTRK 219
Sk 139 ATPFPVQVSSSDVMTMNSVGRTTFEVSASNN...AFVQFG....QFFGGTL....PDEIDAIEVTFKPRRG 198
Ab 151 IHPLKTKLTKKQAMKLNFIGTTRIQIDGNSS...TGREFK....EFFLGSTNPNDMEIIESLEIIIKPAPR 214
Sa 140 LMPLLKQSTREEVLSMSVVGRSSVQINKESS...FFDDLR....SVIGGSV..EEFEDVDSLELVLKPRRK 201
Ap 140 LNPLLTQSTKADAMKMDFIGRSVIQVTKENS...FYEDIR....NFFNGTS..EEFADVDSFELTIKPRRK 201
Ha 140 LHPFMQEATFADALQMPFIGRSSVQVSKENK...LYEDIR....DFFGGTA..EEFAEVDSFEVTVKPRKR 201
Gm 140 PQALLYQATKEEALSLPHIGRTTIELSKENS...FVQDIL....ASISANT..TDTIELDGIEIVIKPKSR 201
Pf 144 ISPLIKQATKEEAVSMEYIGRTTIEVTRDNS...LARHLL....NFLAV.K..SGTDELDSIEITIKPKYG 204
Ps 140 ITPLLKQATKSEAVSMEYIGRTTIEVGQKNT...LTNHLL....NFLSV.G..DCIEELDSIEITIKPKRN 200
G140 T141 T142 K143
D168

```
BP λ RexA        α7                                  β11         TT    β12          TT
BP λ    196  R.DIKKVAKDIIFNPS.........PQFSDISLRAK.DEAGDILTEHYLSEKGHLSAPLN.KVTN.AEIAE  253
BPSfI   196  R.DIKKVAKDIIFNPS.........PQFSDISLRAK.DEAGDILTEHYLSEKGHLSAPLN.KVTN.AEIAE  253
Pbs     196  R.DIKKVAKDIIFNPS.........PQFSDISLRAK.DEAGDILTEHYLSEKGHLSAPLN.KVTN.AEIAE  253
EcK12   196  R.DIKKVAKDIIFNPS.........PQFSDISLRAK.DEAGDILTEHYLSEKGHLSAPLN.KVTN.AEIAE  253
Kp      196  R.NIKQLAKDIVSNPN.........PQFSDISLRAR.DEAGDILTDHYLSEKGHLSASLD.KVTN.AEIAE  253
Cd      194  R.NIKKLTQDIVSNPN.........PQFSDISMRAK.DEAGDILADHYLSEKGHLSASIE.RVKN.AEIAE  251
Kl      197  K.NIKDTISPTLNNLP.........AGIREMTIAAK.QALGDQAIELHVATTGGIYDIVN.PKGN.TSIQA  254
Ga      192  K.NIKKLTQYLIENKM.........NEIEKMKFMAK.EEMADHAVELYLDENILCD.HVD.HKVS.SEIPF  248
Seef    197  R.DIKNLTQDIVNN.D.........ENLSDISMRAK.EEAGDILTDHYLSEKGHISSEIE.KSTN.EDIAN  253
Er      197  R.NIKSLTKEIVNNPN.........KQYSDVAMRAK.DEAGDVLADHYLTEKGHLSATIH.KATN.AEIAE  254
Yr      198  R.DIKTLTKDIVSNQD.........KSYSDVSMRAK.EEAGDILAEHYLSEKGHLSVNLE.KSTN.AEIAE  255
Mo      196  K.DIKAMASDIINNPD.........NQFSDVHIRAK.EHLGDQLTDFYLSGQGVISANLK.KTTN.AELSM  253
Ec1     197  R.NIKSVAKGIISNVD.........EQHESIHLKGK.EQAADILTEYYLSDKGHIGANIY.KSSN.EDIAS  254
Ko      197  K.NIKSLATEIIKNVD.........EQHESILLKGK.EEAADLLTDFYLSSKGHIGANLY.KSSN.DELAS  254
Op      197  R.NIRDMSKEVIRNSE.........GSHSDIHLKGR.EMAADILTDFYLSEKGHLSANIY.KSSS.EEIAE  254
Ye      197  R.NISEMSKEVIRKSD.........DNHNDIHLKGK.ELAADILTDFYLSEKGHLSANLY.KSSS.EEIAE  254
Ia      195  R.NISNMSKELIRNAD.........DGHSDIHLKAK.EYAADLLTDYYLSNKGHISANIY.KSTN.EEIAE  252
Ec2     195  R.NISNMSKELIRNAD.........DSHSDIHLKAK.EYAADLLTDYYLSSKGHISANIY.KSTN.EEIAE  252
Seec    195  R.NIRDMAKELIKNAD.........DSHSDILIKGK.EQAADLLTEYYLSNKGHLAANLY.KSTN.EDIAE  252
Sees    247  R.NIRDMAKELIKNAD.........DSHSDILIKGK.EQAADLLTEYYLSNKGHLAANLY.KSTN.EDIAE  304
Cw      195  R.NISDMAKQLILNAD.........ENHSDILIKGR.EQAADLLTDYYLSNKGHLAANLY.KSTN.EDIAE  252
Ae      197  K.NIQKLTKEILAASY.........DKIDDIKIKGK.ESAADKLTEYYLDGKGHIGAKIIKKSSN.ADIAE  255
Pm2     197  K.DIKHLTKEIINNNY.........DQIDDISIKAK.NSAADILTEYYLDGKGHLGINLPKKVSN.AEIAN  255
Pm1     197  K.NIKGMTKEIISSSY.........GEVDGISIKAK.ETAADLLTEYYLDGKGHVGCNLPKKASN.AEIAN  255
Mm      197  K.DIKGISKEIISSTY.........DSIDQINIKAR.ENAADVLTEYYLDGKGHVGCNLPKKSSN.AEIAN  255
Pc      198  K.DIKGITKDIITNKN.........NSFSDVHIKAR.EEAADILTEYYLSGKGHLGANLY.KASN.ADVAE  255
Xb      196  R.DIKQITTQIIERQG.........DAFSDIHIKAR.EEMADILTEFYLSGKGQISANLY.KVSN.AEIAE  253
As      204  ..NIKEALKKIINKKDK........SGIVRIGAKAKHSELEGQLMDYWLDNENALSDKLS.PSAKRKSLAD  263
Vm      204  ..NMRDVFRHIVHRHDTTGK.SNNTDGIMQIGAKAKHDEFKGQLMDHWLDNENNLTDSLN.PKAKRRKLPD  270
Fa      202  KSNLKKDLQNIAINIPD........SDIDELDARGK.IEATDRMRDLYIHGSGGLKDFIR.PRYE.HEIPS  261
Wc1     203  K.NILEQTKSILDAAT.........SQTDKFILSAK.ANMADNLRDYYIEAAGGLSDTIY.QGKKASDTER  261
Wc3     202  E.SIDEATSYLL.EEY.........VDADKFIIRGK.QDLADNLRDFYINESGVLSDTIE.KSKKDYDTNS  259
Ig      222  T.SIKDEYLALLKSALDSDSEDPSSSGFIDGRIRAR.KDLESRSMDYYINSQANLTDRIK.ITTS.KTIGV  288
Wc2     220  G.NIKGIVMPLLSENHL........NDVEKFNLRAK.EDLEDRLSDYYIMSKGALSDLID.LNGN.GGISQ  278
Sk      199  C.DIKEHIPALTNVISG........DGLEKYMVRAK.ESLDESLSDFYIAGNGFINDYLE.SS.E.MSIAT  256
Ab      215  K.PLTDTIKHKIMTLDD........EGVKGIVLRAK.QNLEDNIKDYYIHSAGGLYDEIT.NTEE.SKILD  273
Sa      202  K.DIKEASGRLLEKVPD........KGLEKMIIKAK.DENGAQLVDMYLAGKGIVSDQID.PTKA.ESLRS  260
Ap      202  Q.NIEPAVKKVIAGVRD........EGLDKMMIRAR.EEIGDEIMDVYLAGKGHVSDNII.KTKD.RTIHE  260
Ha      202  Q.NIDAAMKKIINSVDQ........SGLDKFICRAK.EDLGDHLMDMYLVGQGMISDIIA.KGAD.HEIYE  260
Gm      202  K.NIKPTVSKFLSSIPD........DGVERMIMKAR.NEGASQMTDLYLVGRGAISDSVD.KSQE.TRIPS  260
Pf      205  R.SVKPIVEALISETSD........EGLKKLILKAK.NDASSAMLDLYVAGRGVISDTLD.VADE.LSISS  263
Ps      201  R.NIKPIIEALIENTSD........DELKKLHLKAK.NDATSAMLDLYVAGRGVISDSVE.VIDE.SLIAA  259
```

D215, R219, K221

239-244

```
BP λ RexA     α8
BP λ    254  EMAYCYARMKSDILECFKRQVGKVKD............................................  279
BPSfI   254  EMAYCYARMKSDILECFKRQVGKVKD............................................  279
Pbs     254  EMAYCYARMKSDILECFKRQVGKVKD............................................  279
EcK12   254  EMAYCYARMKSDILECFKRQVGKVKD............................................  279
Kp      254  EIAYCFIRMKTAILESFDRQVGKLKE............................................  279
Cd      252  EMSYCFLRMKASILSSLERQVGKINS............................................  277
Kl      255  QMTTNFINNADLRAAGY.....................................................  271
Ga      249  AMESRYQNSKKIIKELLKQ...................................................  267
Seef    254  EMHSCFTRMKGDINRNLKRQIGVIHRQDNT........................................  283
Er      255  EIEYGFARMKKNILEVFANQVGKLK.............................................  279
Yr      256  EISYCFTRMKVTIMDSFSRQIGDILKQ...........................................  282
Mo      254  EMENCYERNKNAIMKAFDESIPDGLSEI..........................................  281
Ec1     255  EMSTCYLRMKLIIMESYKRTFGDELPL...........................................  281
Ko      255  EMSTCYIKMKPIIAESYNKMFGDELSF...........................................  281
Op      255  EMEACFIRMKPYISKSYQEYFEIEG.............................................  279
Ye      255  EMESCFIRMKPYILKSYKDNFENEISE...........................................  281
Ia      253  EMDICFIKMKNVIMKSYKDFCEDE..............................................  276
Ec2     253  EMEICYIRMKNVIMKSYKDFCEDE..............................................  276
Seec    253  EMETCFIRMKSVIMKSYNDAFDSEL.............................................  277
Sees    305  EMETCFIRMKSVIMKSYNDAFDSEL.............................................  329
Cw      253  EMETCFIRMKAVIIKSYKDHFDGEL.............................................  277
Ae      256  EILATYIRLKPSILESFRKIECEL..............................................  279
Pm2     256  EIYTAFIRLKPAIMESYKYIKEDLE.............................................  280
Pm1     256  EIYTAFAKMKPIILESYENINNELLP............................................  281
Mm      256  EIYTAFIRMKPVIMESYKEIKEELS.............................................  280
Pc      256  EIQYCFIRMKTSIIKSFDQTLGDELKQT..........................................  283
Xb      254  EIHNCFVRMRSAIMRSFTQAVGEDLR............................................  279
As      264  QIGEKFDSNAQLQTLYNSFISNLTNAKTDIDANLVSANDQNTFDDLGGGNEKDGNQKA............  321
Vm      271  QICEKFDENMQLLPLFNAYVSNNRLKEKSVPLLEQYKKVKPFAAKVSKVDTSKEADNDDNVVPLEETEKA  340
Fa      262  SITKAASLNTALQAKIKEFSGDPNYIKTSDPHAIGLNW................................  299
Wc1     262  KIETKILTNTDLKQILDRKRKNEKKQTEDISVITNYITNNRWV...........................  304
Wc3     260  KIVETILRNPSLQDVLTRSKRNDKGKEHNIHSITNFISSNTWCN..........................  303
Ig      289  IINEKIENNSELKERVLSYKQELTYNENQQSRSVQVDIFNKYCDAGGWVITSNCI...............  343
Wc2     279  GIMDALNENEQLASRLLEWENEGNKTYKDSWSLSVYSSHDFWNDFNLSI.....................  327
Sk      257  RIAEKKLANQPLSLKLAEYRNDERYSNDRLEDVASYCNASAWDRFALPSESGDIEASAGEAS........  318
Ab      274  EIKAKIASNSELEVKVNEYEEEIPELLDHRISNFSKSDNWSSD...........................  316
Sa      261  EIRKKKKENAVLKDKVIEHFKNEKIKKASPKVLAKLSDASAWPR..........................  304
Ap      261  QIKEKIKTNSTLQQKVVEHESNSEFKKKEPNSFAKLHDADSWAHRISNL.....................  309
Ha      261  AISAKVGDNDELTAKLGEHDGHEGFKKDEPEVFSGFNNVDAWADKLSDL.....................  309
Gm      261  LLEEKMNRNRFLQDKLTEYRADEGFEENSLDVIVRFTDASAWADLLSDVLQDSSDRPELVDEPDRTEE..  328
Pf      264  VIEDKISKNSILQEQLMEYTNNGQVDKADFSRILRYSDEPAWTAFVDNLPSDYKLQ..............  319
Ps      260  SIEEKIVKNTTLQEQLVEHLRNGQKDKADFSRILRYCDEHSWTAFVDSVQNDYKLRQ.............  316
```

Supplementary Figure S6. Sequence alignment of putative RexA homologs. Sequence alignment of RexA homologs with the secondary structure of the bacteriophage λ RexA mapped above. Colored bars beneath the alignment denote structural segments as follows: globular domain, green; dimerization domain, blue; hinge loop, magenta; swivel loop, red (see **Figure 1**). Positions of conformational mutations are marked below (see **Figures 4** and **5**, **Supplementary Figure S2**). Red text bounded by blue border denotes 70% sequence conservation. Abbreviations are as follows with accompanying NCBI accession numbers or KEGG IDs (6) : BPλ, bacteriophage λ RexA (vg:3827058); BPSfI, bacteriophage SfI gp47 (vg:24722216); Pbs, *Paenibacillus sonchi* (pson:JI735_34930), EcK12, *Escherichia coli* K-12 BW2952 (ebw:BWG_3702), Kp, *Klebsiella pneumoniae* (WP_117261707.1); Cd, *Cedecea davisae* (WP_202303730.1); Kl, *Klebsiella* multispecies (WP_071995728.1); Ga, *Gilliamella apicola* (WP_065635067.1); Seef, *Salmonella enterica* subsp. enterica FNW19H96 (EDW1732907.1); Er, *Erwinia* sp. S38 (WP_200545456.1); Yr, *Yersinia ruckeri* (WP_234057212.1); Mo, *Morganellaceae* multispecies (WP_154640079.1); Ec1, Escherichia coli (WP_253764069.1); Ko, unclassified *Kosakonia* multispecies (WP_200133690.1); Op, *Obesumbacterium proteus* (WP_234559868.1); Ye, *Yersinia enterocolitica* (WP_050128085.1); Ia, *Izhakiella australiensis* (WP_096777782.1); Ec2, *Escherichia coli* (WP_197940034.1); Seec, *Salmonella enterica* subsp. *enterica* serovar Cubana (seec:CFSAN002050_11670); Sees, *Salmonella enterica* subsp. *enterica* serovar Saintpaul (ECA2934630.1); Cw, *Citrobacter werkmanii* (WP_085048607.1); Ae, Arsenophonus endosymbiont of Apis mellifera (aet:LDL57_11880); Pm2, *Proteus mirabilis* (WP_143474652.1); Pm1, *Proteus mirabilis* (WP_206081156.1); Mm, *Morganella morganii* (WP_049246396.1); Pc, *Photorhabdus cinerea* (WP_166310405.1); Xb, *Xenorhabdus bovienii* (WP_038244014.1); As, *Alteromonas stellipolaris* R10SW13 (aaw:AVL56_04330); Vm, Vibrio mimicus (vmi:AL543_00300); Fa, *Frateuria aurantia* (fau:Fraau_1963); Wc1, *Wohlfahrtiimonas chitiniclastica* (WP_213398763.1); Wc3, *Wohlfahrtiimonas chitiniclastica* (WP_094493134.1); Ig, Ignatzschineria sp. HR5S32 (ign:MMG00_12050); Wc2, *Wohlfahrtiimonas chitiniclastica* (WP_213405574.1); Sk, *Shewanella khirikhana* (skh:STH12_00053); Ab, *Acinetobacter baumannii* BJAB0715 (abab: BJAB0715_02483); Sa, *Salinisphaera* sp. (MBS61511.1); Ap, *Abyssibacter profundi* (WP_109719971.1); Ha, *Halomonas* sp. 3F2F (WP_226930571.1); Gm, *Gallaecimonas mangrovi*

(WP_115720386.1); Pf, *Pseudomonas fragi* (pfz:AV641_12615); Ps, *Pseudomonas syringae* (WP_198722127.1).

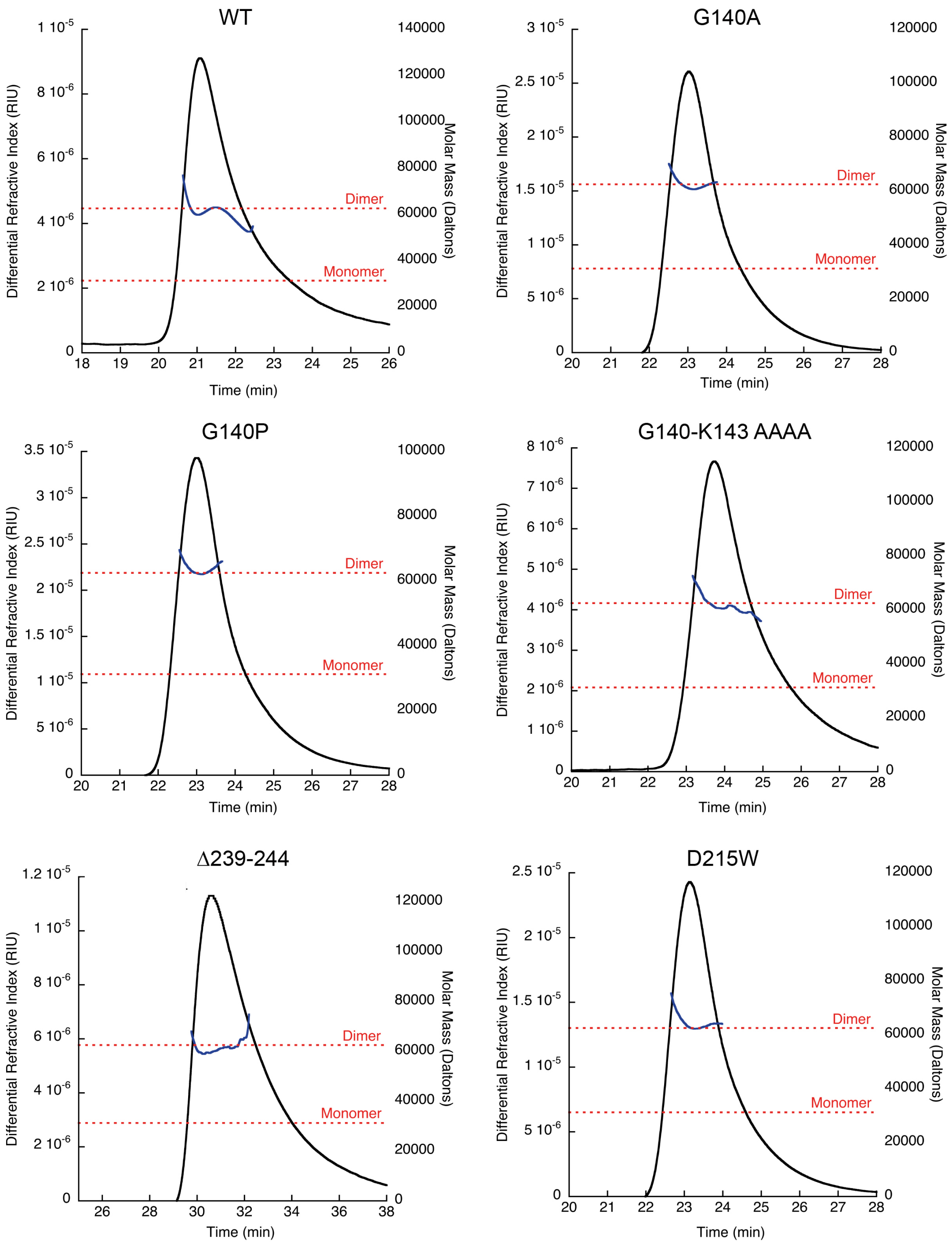

Supplementary Figure S7. SEC-MALS analysis of RexA mutants. Black line denotes differential refractive index and blue line denotes measured mass across each peak. Dashed red lines indicate the predicted molecular weight of a RexA monomer and dimer.

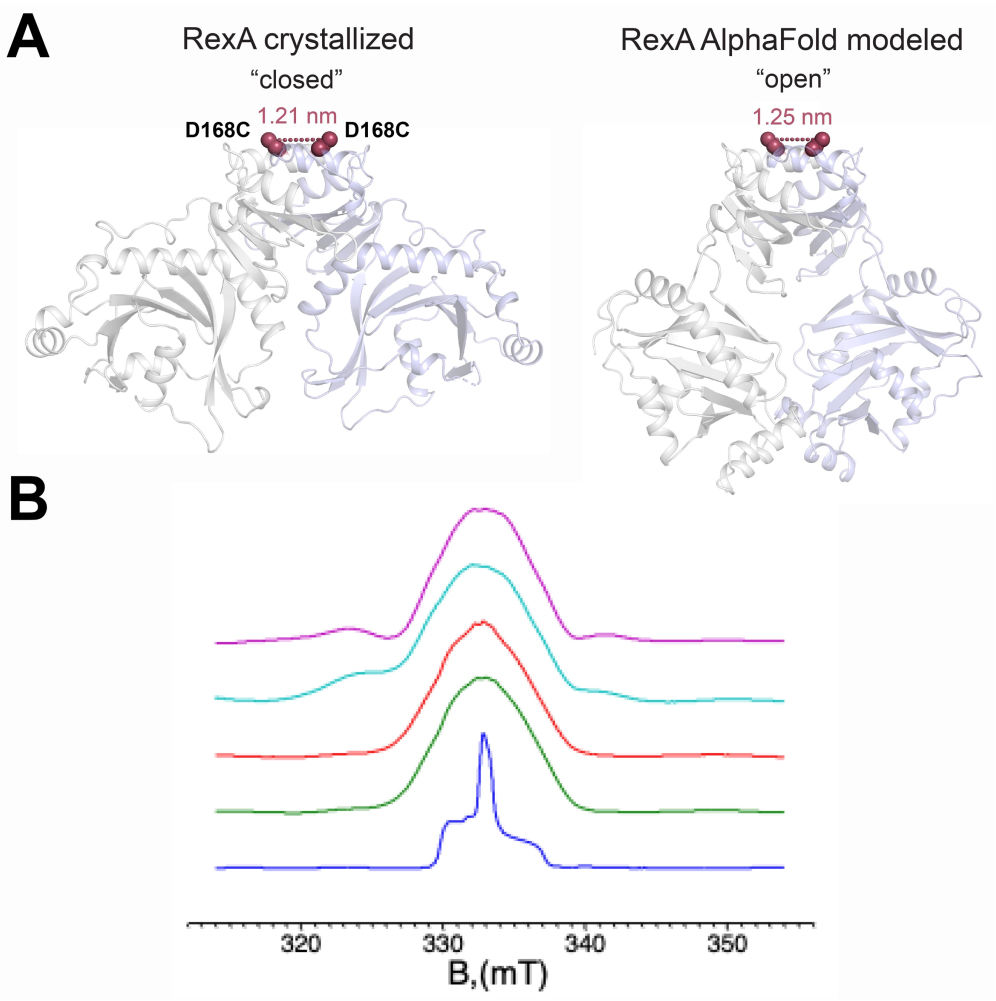

Supplementary Figure S8. ESR spectroscopy measurements for D168C. A. Predicted distances between the D168C substitutions (red spheres) in the crystallized (closed) and AlphaFold modeled (open) RexA dimer structures. B, Integrated CW-ESR spectra of D168C (green); D168C/DNA (red); D168C/D215W (cyan); and D168C/D215W/DNA (magenta) spin-labeled RexA mutants. The rigid-limit unbroadened nitroxide spectrum (blue) was plotted as a reference. All D168C spectra show large dipolar broadening ~2 mT corresponding to ~1 nm distance between spin labels. Spectral features caused by nitroxide labels not having partners were subtracted out. There is no visible effect of DNA but D215W mutation adds shoulders to the spectra.

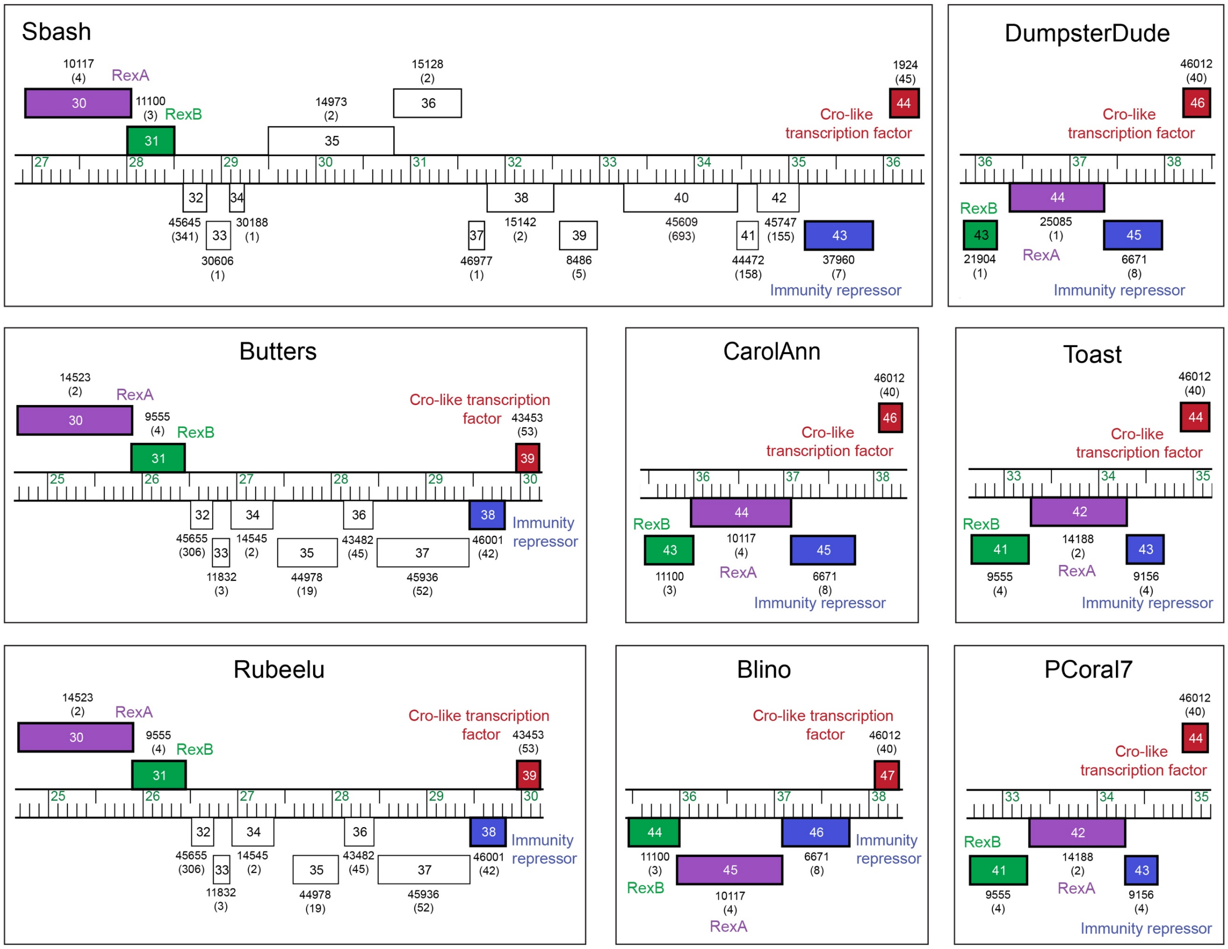

Supplementary Figure S9. Gene neighborhoods surrounding RexA-like genes in Actinobacteriophages. Genomic organization around RexA-like genes in Sbash, DumpsterDude, Butters, Rebeelu, CarolAnn, Blino, Toast, and PCoral7 phages generated via Phamerator (7). Individual genes are depicted as boxes with the gene name inside the box and the Phamily designation and number of Pham members (in parentheses) shown below each box. Genes corresponding to RexA (purple), RexB (green), a CI-like immunity repressor (blue), and Cro-like transcription factor (red) are colored and labeled.

```
λ RexA              1 : --------MKNGFYATYRSKNKGKDKRSINLSVFLNSLLADNH-HLQVGSNYLYIHKIDG-------KTFLFTKTNDKSLVQKINRSK :  72
Sbash gp30          1 : -------------------MTGERQIVAYGLRVEVDGRAQRRTKNPPPHTPYPANFNGAGADLLMFFSSFVNALPTD----------- :  58
CarolAnn gp44       1 : -----------------MGTWGDRRIVLYTLRVALDGRVFRTS--AAAGEPFVANFNGSGADLLHFLSSFIEALPTD----------- :  58
Toast gp42          1 : -------------------------MPSYGIRFYKVELFNGNK-----QTPLSFIE-EDGGKSWRYGE-HLATVLE------------ :  44
DumpsterDude gp44   1 : --------------------MPRRTMTVTEVWAHPVG--SRKE-----KDRVDLTALPDGMDLLHAFYGFAADVDAR----------- :  50
Butters gp30        1 : MLWDRTSHVLSISYANLRTSTHPPLMTNYGFRLFQVGVYRNGR-----GDPCAVDA--IGDDKQHFRD-YLFDLAN------------ :  68

λ RexA             73 : ASVEDIKNSLADDESLGFPSFLFVEGDTIGFARTV-----------------------------FGPTTSDLTDFLIGKGMSLSSGER : 131
Sbash gp30         59 : ------KLIRRGDRHFGIPEEVERLGRTI-------------------------RLRLSGGESGRRSKIT------------------ :  97
CarolAnn gp44      59 : ------KHIERDERHFGQPTSIRRRGRTI-------------------------SWRMDGGESGRKSQIR------------------ :  97
Toast gp42         45 : --------RFKENQVRGFPRSQTPDGESVDQ--------------------IRKKTAMVFNAVDRRGPNTVVAEYRVGRSDDFDR--- : 101
DumpsterDude gp44  51 : ------QLLKRENESFASVVSLEEKGRAV-------------------------TLAVDVGRFGERGTIT------------------ :  89
Butters gp30       69 : --------RKKDCVVHGSPPRSKEEGDSDESADVDDHESVDDDAEQQPTGAKRPRPVLVITDVSFRGD-HVVIEYLYGRDLGYTH--- : 144

λ RexA            132 : VQIEPLMRGTTKDDVMHMHFIGRTTVKVEAKLPVFGDILKVLGATDIEGELFDSL---DIVIKPKFKRDIKKVAKDIIF--------- : 207
Sbash gp30         98 : ------LKAGGKEETREPSGVEWEPFRVAIVIPKDFNQ----GWLLVEKSGYHSV---PTEWRQELVRAFKAAYPDYVL-KISSITEM : 171
CarolAnn gp44      98 : ------LRRDADAQERDRSGVEWEPFWAFAVVPENSVV----GWLLVEKAGRHSL---PTEWRKELQRQFAGAYRGYRL-EIGIVREM : 171
Toast gp42        102 : --AYPAPDLETSDYLELNGYAPARPYRAVLMVPPEGEV----GMLAVEAIGRTCPYEFFTKWATKWSRDYQAELD---SSDPLDDDKV : 180
DumpsterDude gp44  90 : -----DVTTMIERDRFTRTDAISVITHGIFTLPKGCTS----ALVFIERSGNQSG---IIRVLELFQHQFRLAYPDLIL-ETTAVVES : 164
Butters gp30      145 : --AGYSEQDTTAKAVRIANLKSVRPYRSVFMFPDVGNS----GVVAVEDASRAHASKKLEQWLKAWAREEAHAVAEIKRKETGKKSIK : 226

λ RexA            208 : ------NPSPQFSDISLRAKDEA----GDILTEHYLSEKGHLS--APLN---------KVTNAEIAEEM--AYCYARMK--------- : 263
Sbash gp30        172 : SLWSQVE--NSN-EPRLLAVEVIMRSS----DSSGDHSAGHPANMATYDTHVWEAVHAQPG--RVLRQLRRRFTRRKTK---DGL--- : 244
CarolAnn gp44     172 : SLWAQVE--NALDEERLIGFEVAYRSA---STTAGSGAAGYERGMEKYERRMWRAPADQPKKGRKLREFRRAHT-KTLI---EGT--- : 247
Toast gp42        181 : RAWWRLKPTPLGSKSQLVDFIKGGTIEELILINHYIDKTRNER-QERFR------------VKAKVRTGQRPWAKKRLNEV----FEV : 251
DumpsterDude gp44 165 : EAWLSYA--------SLVKVSAHRRA-------SGSDEA--DNNSKRIKRSEYGELAHTLVPARGERTLPR-FLYDRLV---AGA--- : 228
Butters gp30      227 : PVWWSMRFTPLSDPERLAALMKNGTSSKIVLTKQGGSDARTPG-RSPLK---VEMGLDEPASIAKARRLITGWLPKFNRTPQAGDAQV : 310

λ RexA            264 : -----------------------SDILECFKRQVGKVKD------------------------------------------------- : 279
Sbash gp30        245 : ---------VEITQPLDVDDLSDDDAKIELKDDVRQIKARVLNSEGRKKTVIFGGRE-PTMTIVMDGVFDLSPSATKFWQEARSA--- : 319
CarolAnn gp44     248 : ---------REIELPFEADDLSDDRYTIRLRDDVAEIKATLYNSEGQPKTVVFEGLD-PQQTIVMEDTALAPPDQDRFDTECRSA--- : 322
Toast gp42        252 : DSDEVFARQLAENFGNSVEHLDIDDGYVVVRTEA-GVKNIS--PSRMPEVFTYPVSD----------DR--PT-MEEFLTAV------ : 317
DumpsterDude gp44 229 : ---------LKPSELLDFDE-SDE---------AAHVE-VTLEHNGQRKTFLLGHEKRPSISYLL------SNHGEDEWSLERVRDYA : 290
Butters gp30      311 : AANRELAAVLADNYQ-GFDEEDYDDAWIEVKDAAGKAKKIS--PSRWADIFIYPVRN----------STECPP-PALFYRRV------ : 378

λ RexA              - : --------------------------------------------------------- :   -
Sbash gp30        320 : ----VVDLATSGGVSLAPKWDTGEWEHPENAIKVEVSLTDEPDQDDAASAGGTGTTA : 372
CarolAnn gp44     323 : ----VRDLAASNMVGLAPKWDTGDWVHPEDAVKLEVKSDAPSPERDQGSEAN----- : 370
Toast gp42        318 : -KRHAIPLAKVID----AQVDFDSW-------------------------------- : 337
DumpsterDude gp44 291 : FDTHADTIYGRLGWEWTPAHTVGAWTDSQRGARLVVRGGEQS--------------- : 332
Butters gp30      379 : -QESVRPLEKSLE----LSIDWAGWGGDSG--------------------------- : 403
```

Supplementary Figure S10. Sequence alignment of unique RexA-like proteins present in Actinobacteriophage viruses. Alignment includes unique RexA homologs present in the Actinobacteriophage Database (PhagesDB) (8). Sequences for Blino gp45, PCoral7 gp42, and Rebeelu gp30 are omitted as they are each 100% identical to CarolAnn gp44, Toast gp42, and Butters gp30, respectively. Sequence shading indicates conservation: white text on black background, 100% conserved; white text on dark gray background, 80% conserved; black text on light gray background, 60% conserved.

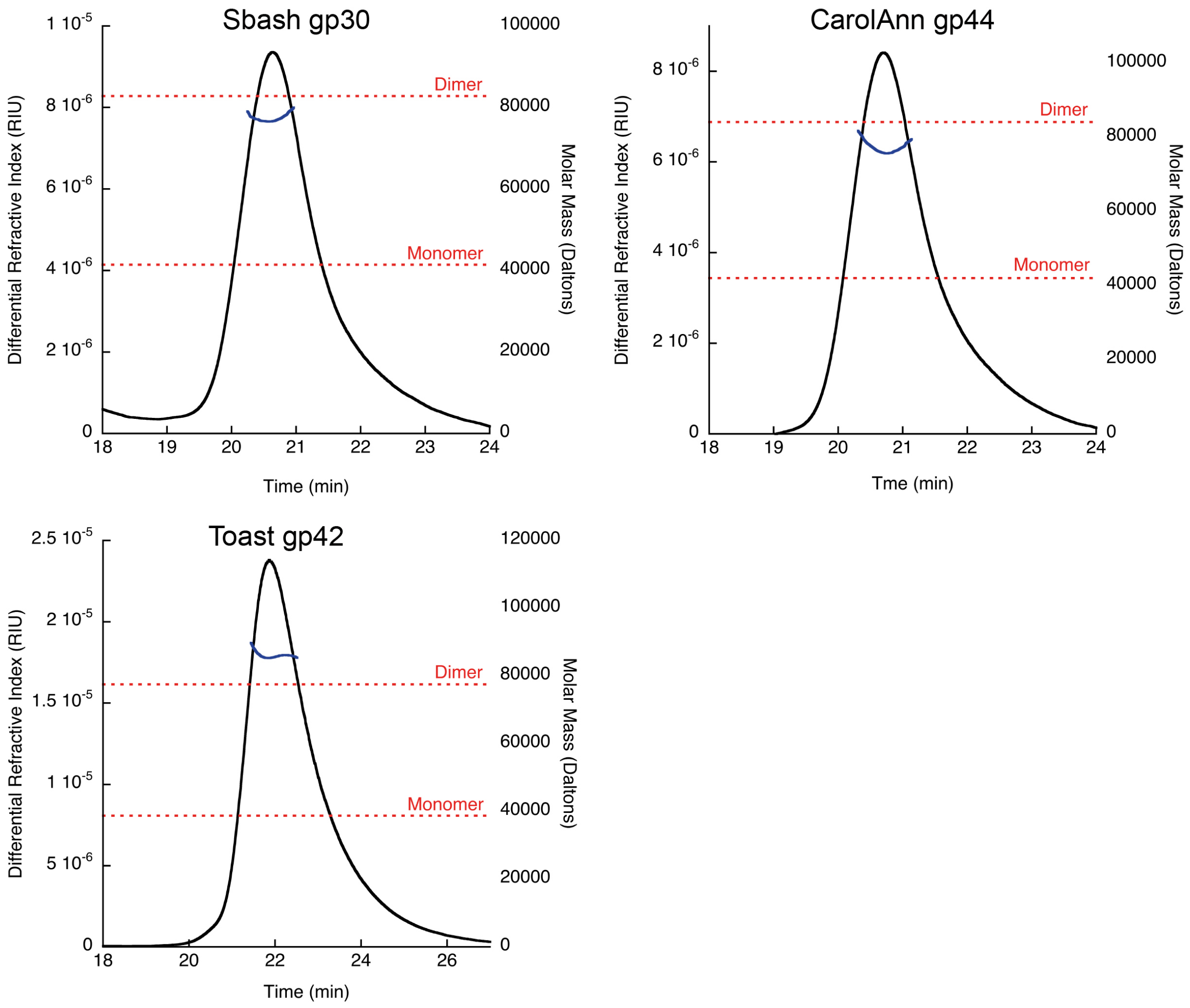

Supplementary Figure S11. SEC-MALS analysis of purified RexA homologs. Black line denotes differential refractive index and blue line denotes measured mass across each peak. Dashed red lines indicate the predicted molecular weight of a monomer and dimer for each homolog.

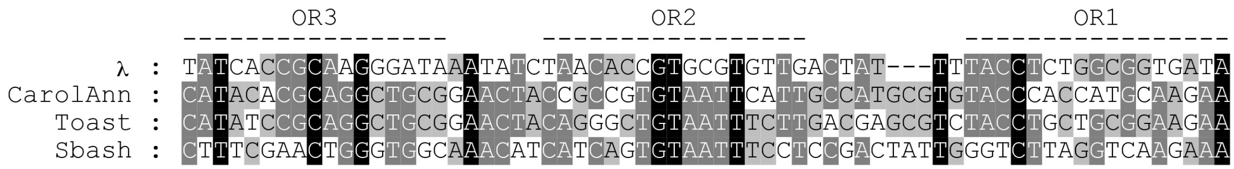

Supplementary Figure S12. Comparison of operator sequences used in binding experiments. Alignment of the genomic region encompassing the operator sites O_R1, O_R2, and O_R3 in phages λ, CarolAnn, Toast, and Sbash. Sequence shading indicates conservation: white text on black background, 100% conserved; white text on dark gray background, 80% conserved; black text on light gray background, 60% conserved. DNA substrates containing λ OR1 and OR2 were used for EMSAs, limited proteolysis, and ESR experiments (**Figures 4, 5,** and **7**) while substrates containing OR1, OR2, and OR3 were only used for EMSAs with individual RexA homologs (**Figure 7C**). See **Supplementary Table S2** for sequences of each individual substrate.

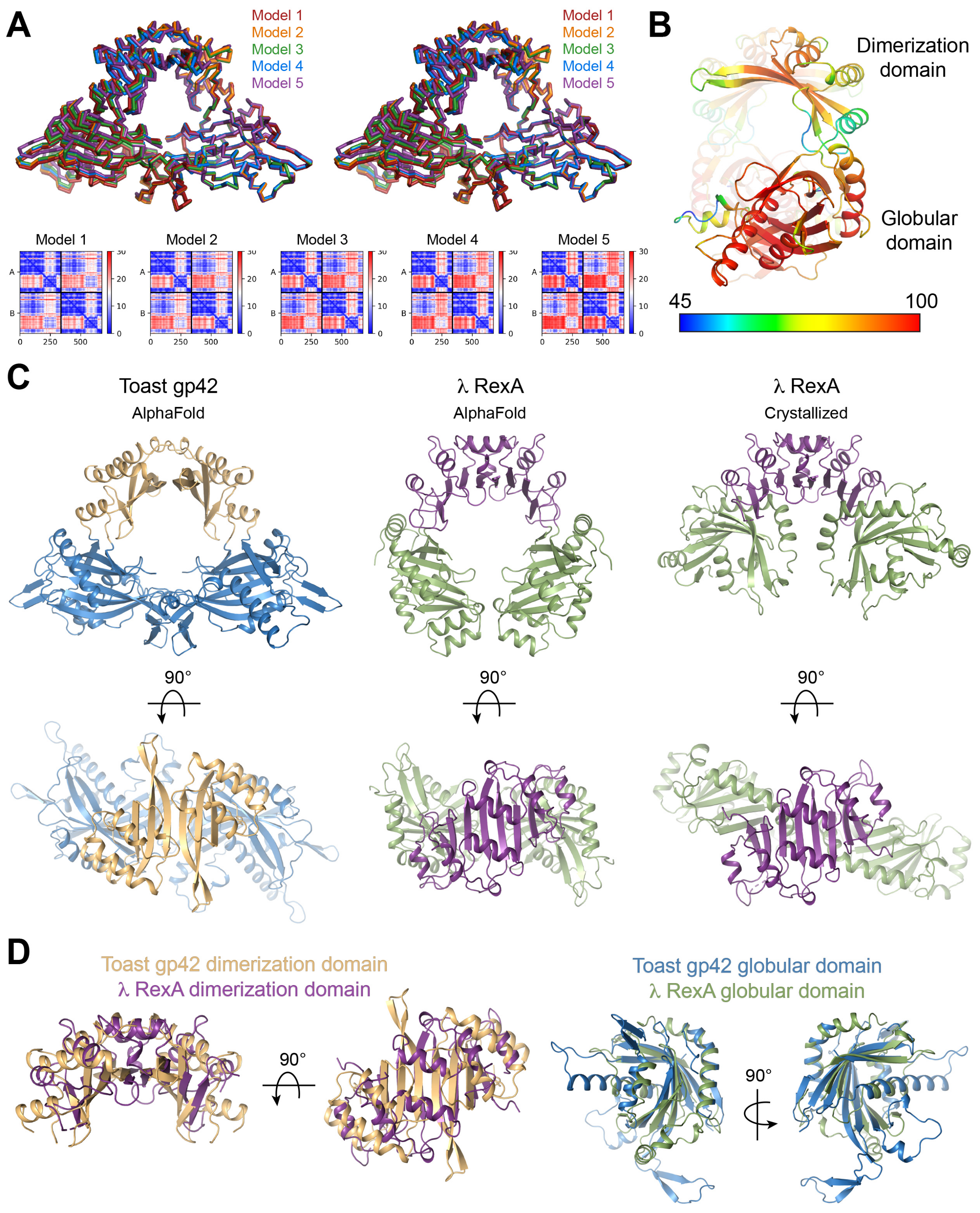

Supplementary Figure S13. AlphaFold model of Toast gp42 and comparison to RexA. A. Superposition of top 5 Toast gp42 models generated with AlphaFold-Multimer (9) viewed in

stereo. Predicted aligned error plots are shown below. B. Side view of Toast gp42 model colored according to the predicted local distance difference test (pLDDT) score (0-100), with values greater than 90 indicating high confidence and values below 50 indicating low confidence. Scale bar denotes per residue confidence coloring for pLDDT scores ranging from 45 to 100. C. Comparison of Toast gp42 AlphaFold model (left) with the RexA AlphaFold model (center, predicted open conformation) and crystallized RexA structure (right, closed conformation). Side and top views are shown for each. Coloring as follows: Toast gp42 dimerization domains, light orange; Toast gp42 globular domains, sky blue; RexA dimerization domains, violet purple; RexA globular domains, smudge. D. Superposition of the dimerization domains (left) and individual globular domains (right) from the Toast gp42 AlphaFold model and the crystallized RexA coordinates.

SUPPLEMENTARY REFERENCES

1. Thomason,L.C., Morrill,K., Murray,G., Court,C., Shafer,B., Schneider,T.D. and Court,D.L. (2019) Elements in the λ immunity region regulate phage development: beyond the 'Genetic Switch.' *Mol Microbiol*, **112**, 1798–1813.

2. Thomason,L.C., Schiltz,C.J., Court,C., Hosford,C.J., Adams,M.C., Chappie,J.S. and Court,D.L. (2021) Bacteriophage λ RexA and RexB functions assist the transition from lysogeny to lytic growth. *Mol Microbiol*, **116**, 1044-1063.

3. Hochschild,A. and Lewis,M. (2009) The bacteriophage λ CI protein finds an asymmetric solution. *Curr Opin Struc Biol*, **19**, 79–86.

4. Oppenheim,A.B., Kobiler,O., Stavans,J., Court,D.L. and Adhya,S. (2005) Switches in bacteriophage lambda development. *Annu Rev Genet*, **39**, 409–429.

5. Casjens,S.R. and Hendrix,R.W. (2015) Bacteriophage lambda: Early pioneer and still relevant. *Virology*, **479**, 310–330.

6. Kanehisa,M., Furumichi,M., Tanabe,M., Sato,Y. and Morishima,K. (2017) KEGG: new perspectives on genomes, pathways, diseases and drugs. *Nucleic Acids Res*, **45**, D353–D361.

7. Cresawn,S.G., Bogel,M., Day,N., Jacobs-Sera,D., Hendrix,R.W. and Hatfull,G.F. (2011) Phamerator: a bioinformatic tool for comparative bacteriophage genomics. *Bmc Bioinformatics*, **12**, 395.

8. Russell,D.A. and Hatfull,G.F. (2016) PhagesDB: the actinobacteriophage database. *Bioinformatics*, **33**, 784–786.

9. Evans,R., O'Neill,M., Pritzel,A., Antropova,N., Senior,A., Green,T., Žídek,A., Bates,R., Blackwell,S., Yim,J., *et al.* (2022) Protein complex prediction with AlphaFold-Multimer. *Biorxiv*, 10.1101/2021.10.04.463034.
